# A germline-to-soma signal triggers an age-related decline of mitochondrial stress response

**DOI:** 10.1038/s41467-024-53064-0

**Published:** 2024-10-08

**Authors:** Liankui Zhou, Liu Jiang, Lan Li, Chengchuan Ma, Peixue Xia, Wanqiu Ding, Ying Liu

**Affiliations:** 1 State Key Laboratory of Membrane Biology, New Cornerstone Science Laboratory, Institute of Molecular Medicine, College of Future Technology, Peking University, 100871 Beijing, China; 2 Peking-Tsinghua Center for Life Sciences, Academy for Advanced Interdisciplinary Studies, Peking University, 100871 Beijing, China; 3 Bioinformatics Core Facility, College of Future Technology, Peking University, 100871 Beijing, China

**Keywords:** Mitochondria, Stress signalling, Ageing, Tissues

## Abstract

The abilities of an organism to cope with extrinsic stresses and activate cellular stress responses decline during aging. The signals that modulate stress responses in aged animals remain to be elucidated. Here, we discover that feeding *Caenorhabditis elegans* (*C. elegans*) embryo lysates to adult worms enabled the animals to activate the mitochondrial unfolded protein response (UPR^mt^) upon mitochondrial perturbations. This discovery led to subsequent investigations that unveil a hedgehog-like signal that is transmitted from the germline to the soma in adults to inhibit UPR^mt^ in somatic tissues. Additionally, we find that the levels of germline-expressed piRNAs in adult animals markedly decreased. This reduction in piRNA levels coincides with the production and secretion of a hedgehog-like signal and suppression of the UPR^mt^ in somatic cells. Building upon existing research, our study further elucidates the intricate mechanisms of germline-to-soma signaling and its role in modulating the trade-offs between reproduction and somatic maintenance within the context of organismal aging.

## Abstract (web summary)

As worms reach adulthood and commit to reproduction, their ability to respond to mitochondrial stress diminishes. Here, Zhou et al. identify a germline-to-soma Hedgehog-like signal that suppresses UPR^mt^, prioritizing reproduction over somatic maintenance.

## Introduction

Aging, a time-related deterioration in organisms, is caused by changes in the homeostatic regulation of intrinsic processes, which can be accelerated by environmental stressors. Extensive studies in model organisms have demonstrated that during aging, the abilities to cope with different stresses and activate cellular stress responses are reduced^1–6^. Animals respond to mitochondrial perturbation by activating the mitochondrial unfolded protein response (UPR^mt^), a mitochondrion-to-nucleus communication that activates the transcription of mitochondrial stress response genes^7,8^. The UPR^mt^ exhibits a distinct temporal activation profile in *C. elegans*, with the most significant induction observed prior to adulthood, particularly during developmental stages^9^. This suggests that once worms become adults and commit to reproduction, their ability to sense mitochondrial damage or respond to mitochondrial stress is diminished. Similarly, the decline of the mitochondrial stress response has also been reported in aged rats^10^. These phenomena correlate with the observations that mitochondrial disorders occur during aging, and mitochondria play essential roles in age-related diseases such as neurodegenerations.

In this work, we identify a potential piRNA-regulated germline-to-soma Hedgehog signaling pathway that enables the germline to communicate with key metabolic and stress response tissues, prioritizing resource allocation for reproduction over somatic maintenance.

## Results

### UPR^mt^ is activated in adult worms upon ingestion of embryo lysates

To understand the molecular mechanisms that govern the age-related decline of UPR^mt^ activation, we first verified this phenomenon by feeding *C. elegans* with different mitochondrial inhibitors (antimycin, carbonyl cyanide 3-chlorophenylhydrazone (CCCP), and paraquat), *Escherichia coli* (*E. coli*) strains expressing double-stranded RNAs (dsRNAs) that target nuclear-encoded mitochondrial genes *atp-2*, *cco-1* or *spg-7*, or a *Pseudomonas aeruginosa* strain (PA14) reported to inhibit mitochondrial function^11^. The RNAi efficiency for each gene was confirmed by quantitative PCR (Supplementary Fig. 1). Activation of UPR^mt^, detected by expression of GFP driven by the promoter of a mitochondrial chaperone gene *hsp-6* (*hsp-6p::gfp*), was notably observed at the L3 larval stage, but not in day 3 adults (Supplementary Fig. 2a, b). In addition, up-regulation of endogenous UPR^mt^ transcripts was markedly observed in L3 worms, but not in day 3 adults, fed with antimycin (Supplementary Fig. 2c).

**Fig. 1:**
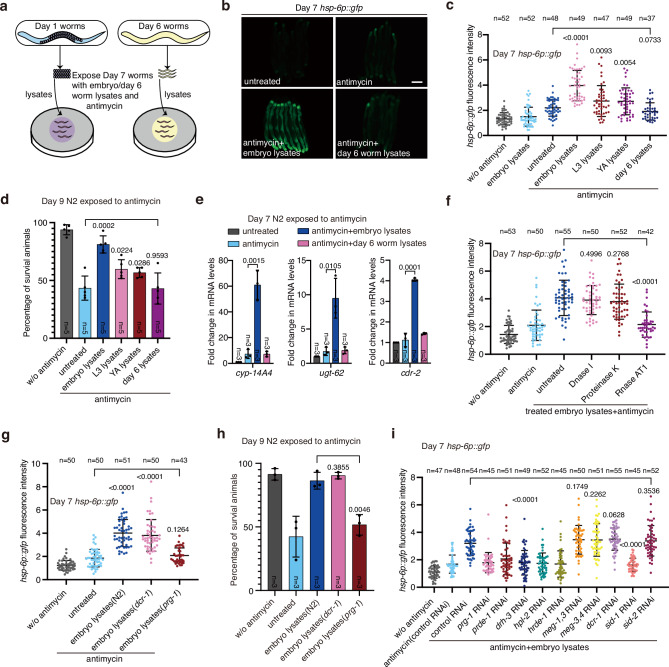
Feeding adult worms with embryo lysates enables UPR^mt^ activation and promotes mitochondrial stress resistance. **a** Schematic depicting the experimental procedure to treat day 7 *hsp-6p*::*gfp* adult worms with antimycin and lysates from embryos or day 6 adult worms. Embryo were harvested from day 1 adult worms. **b** Representative fluorescence images of *hsp-6p*::*gfp* worms with the indicated treatments. Antimycin and embryo/day 6 worm lysates were administered to day 7 adult worms for 24 h prior to visualization. Scale bar, 200 µm. **c** Quantification of GFP fluorescence intensity in panel (**b**). **d** Survival rate of day 9 N2 adults after 72 h exposure to antimycin and embryo/worm lysates. **e** qRT-PCR analysis of the indicated UPR^mt^ genes in day 7 wild-type (N2) worms with the indicated treatments. **f**, **g** Quantification of GFP fluorescence intensity in *hsp-6p*::*gfp* worms with the indicated treatments. Antimycin and embryo/worm lysates were provided to day 7 adult worms for 24 h. **h** Survival rate of day 9 N2 adults after 72 h exposure to antimycin and embryo lysates (from N2, *dcr-1*, or *prg-1* mutant worms). **i**, Quantification of GFP fluorescence intensity in *hsp-6p*::*gfp* worms with the indicated treatments. Antimycin and embryo lysates were provided to day 7 adult worms for 24 h. For panels (**d**, **e**) and (**h**) represents the number of independent experiments; for panels (**c**, **f**, **g**) and (**i**) represents the number of worms. Error bars indicate mean ± SD. *p* values were assessed using a two-tailed *t* test. RNAi treatment started at the L1 stage across all figure panels, unless specified otherwise. Source data are provided as a Source data file.

To identify the genes that modulate age-related decline of UPR^mt^ activation, we then carried out several rounds of ethyl methane sulfonate (EMS) mutagenesis and forward genetic screens in *C. elegans*. Unfortunately, we failed to obtain any mutant worms capable of activating UPR^mt^ in late adulthood. Inspired by previous studies demonstrating that establishing a shared circulatory system between young and aged mice can reverse specific aging phenotypes in older animals through factors present in young mice’s blood^12,13^, we fed *C. elegans* embryo lysates to adult worms to explore similar effects. Interestingly, we found that feeding adult worms with *C. elegans* embryo lysates, but not lysates from adult worms, activated UPR^mt^ in adult worms (Fig. 1a–c and Supplementary Fig. 2d–f). Previous study has indicated that the age of parental worms can affect the traits of their offspring^14^. In light of this, we investigated whether embryo lysates from parental *C. elegans* at day 1, day 2, and day 3 of adulthood would have distinct effects. Our findings revealed that embryo lysates from worms at any of these days of adulthood equally induced UPR^mt^ activation in adult worms (Supplementary Fig. 2g). Feeding adult worms with embryo lysates also promoted the survival of worms treated with mitochondrial inhibitors (Fig. 1d and Supplementary Fig. 2h). In addition, the effect of embryo lysate treatment was not specific to GFP fluorescent reporters, as the levels of endogenous UPR^mt^ transcripts were also elevated upon embryo lysate feeding (Fig. 1e). UPR^mt^ signaling activates transcriptional responses through the transcription factor ATFS-1, in conjunction with the chromatin remodeling protein DVE-1 and its interacting protein UBL-5^8^. Knockdown of *atfs-1* and *dve-1*, but not *ubl-5* in adult worms prevented the activation of UPR^mt^ and the elevated survival of embryo lysate-treated worms (Supplementary Fig. 2i, j). Moreover, we compared lysates from embryos, L3, young adult (YA) and day 6 adult worms. We found that embryo lysates were the most effective in activating UPR^mt^ and promoting the survival of adult worms challenged with mitochondrial insults (Fig. 1c, d). To investigate whether embryo lysates affect a broader spectrum of stress responses in adult worms, we performed feeding assays with ER unfolded protein response (UPR^ER^) and heat shock response (HSR) reporter strains (*hsp-4p::gfp* and *hsp-16.2p::gfp*, respectively). We found that embryo lysates specifically activated UPR^mt^ in adult worms, they did not affect UPR^ER^ or HSR (Supplementary Fig. 3a–d). In addition, embryo lysates marginally improved survival under heat shock (Supplementary Fig. 3e, f).

We next aimed to determine what substance in the embryo lysates enables UPR^mt^ activation in adult worms. DNase-, RNase- and proteinase-treated embryo lysates were fed to adult worms while they were challenged with mitochondrial insults. RNase treatment greatly impaired the ability of embryo lysates to activate UPR^mt^ and promote animal survival upon mitochondrial inhibition. In contrast, DNase or proteinase treatment had no such effect (Fig. 1f and Supplementary Fig. 3g). To further test the type of RNA required for UPR^mt^ activation in adult worms, we fed adult worms with embryo lysates generated from different mutants. Interestingly, embryo lysates, or L3 worm lysates from *prg-1* mutants that lack mature PIWI-interacting RNAs (piRNAs)^15,16^ were not able to activate UPR^mt^ and promote animal survival under mitochondrial stress conditions (Fig. 1g, h and Supplementary Fig. 3h). In contrast, embryo lysates from *dcr-1* mutants, which lack mature microRNAs (miRNAs) and small interfering RNAs (siRNAs)^17–19^, were still capable of activating UPR^mt^ and promoting animal survival (Fig. 1g, h). In addition, RNAi knockdown of genes involved in various piRNA-related processes suppressed the ability of embryo lysates to activate UPR^mt^ in adult worms. These processes include piRNA maturation (*prg-1*)^15,16^, piRNA biogenesis (*prde-1*)^20^, and the biogenesis of piRNA-dependent 22G-RNAs (secondary siRNAs produced when piRNAs interact with target mRNAs), such as *drh-3*. It should be noted that DRH-3 is involved in the production of both piRNA-dependent and piRNA-independent 22G-RNAs, as well as the CSR-1 pathways^21–23^. Other suppressed genes are involved in nuclear silencing (*hpl-2* or *hrde-1*)^24,25^ and dsRNA transmembrane transport (*sid-1*) (Fig. 1i). In contrast, embryo lysates were still capable of promoting UPR^mt^ activation in animals with RNAi knockdown of *meg-1*, *meg-3* or *meg-4*, which are required for cytoplasmic but not perinuclear P granule formation^26,27^ (Fig. 1i). Consistently, embryo lysates from worms subjected to RNAi silencing of genes in the piRNA biogenesis pathway also failed to activate UPR^mt^ and enhance survival under mitochondrial stress conditions (Supplementary Fig. 3i, j). piRNAs are predominantly expressed and function in the germline^28^. Therefore, when fed to a germline-deficient mutant (a temperature-sensitive *glp-4* allele raised under the restrictive temperature^29^), the embryo lysates could not promote UPR^mt^ activation (Supplementary Fig. 3k). These results suggest that piRNAs in the embryo lysates might function in the germline to modulate UPR^mt^ activity in the somatic tissues of adult worms. Through examining a variety of germline mutants^29–32^, we found that the elimination of germline stem cells (GSCs) (*glp-1* or *glp-4*)—rather than oocytes (*fem-3*) or sperm (*fog-1*)—contributes to the disruption of UPR^mt^ in adult worms (Supplementary Fig. 3l).

**Fig. 2:**
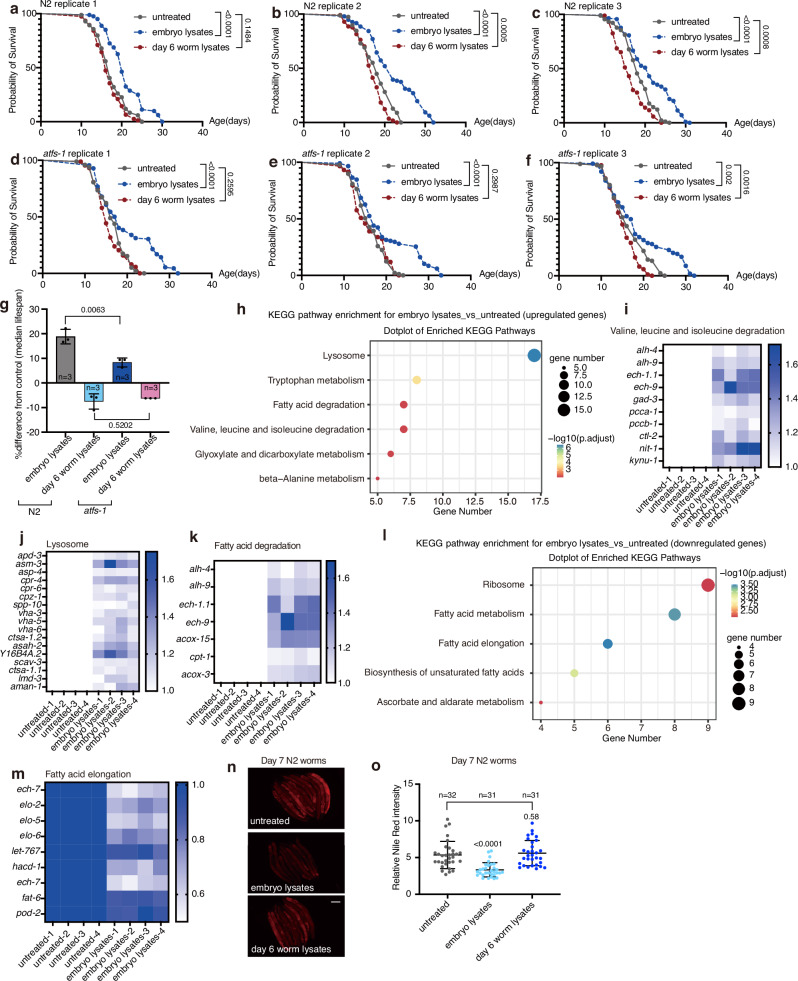
Feeding adult worms with embryo lysates extended lifespan. **a**–**f** Survival curves of wild-type (N2) worms (a-c) or *atfs-1* mutants (d-f) treated with embryo lysates or day 6 worm lysates. Lifespan data from 3 biological replicates are shown. **g** Median lifespan extension of wild-type (N2) worms and *atfs-1* mutant worms treated with embryo lysates or day 6 worm lysates. **h**, **l** Gene Ontology analysis of embryo lysate feeding-dependent upregulated (h) or downregulated (l) genes in day 7 wild-type worms. **i**, **j**, **k**, **m** Heat map plots illustrate the differentially expressed genes that depend on embryo lysate feeding, covering pathways such as valine, leucine, and isoleucine degradation (**i**), lysosome genes (**j**), fatty acid degradation (**k**), and fatty acid elongation (**m**). **n** Representative fluorescence images of adult day 7 N2 worms treated with Nile Red and/or lysates for 24 h. Scale bar, 200 µm. **o** Quantification of Nile Red intensity in panel (**n**). Error bars indicate mean ± SD. *n* represents the number of independent experiments for panel (**g**), and the number of worms for panel (**o**). *p* values were assessed using the Log-rank (Mantel–Cox) test for panels (**a**–**f**), and a two-tailed *t* test for panels (**g**, **o**). For KEGG in panels (**h**, **i**), a hypergeometric distribution test is used to assess enrichment of differentially expressed genes. Detailed methods are in the methods section. For heat maps in panels (**i**–**k**) and (**m**), a two-tailed *t* test is used to evaluate differential gene expression. Source data are provided as a Source Data file.

Mitochondrial function is associated with worm lifespan^33^. Lifespan analysis revealed that adult worms fed with embryo lysates, but not day 6 worm lysates, experienced a ~20% lifespan extension (Fig. 2a–c). This extension reduces to ~10% in *atfs-1* mutants (Fig. 2d–g), suggesting embryo lysates may also regulate lifespan through mechanisms beyond UPR^mt^ regulation. To systematically evaluate the effect of embryo lysates feeding, we conducted RNA sequencing (RNA-seq) on adult worms that were either untreated or treated with embryo lysates (Supplementary Data 1). Our findings revealed that embryo lysates affected the expression of genes related to fatty acid metabolism, degradation of branched-chain amino acids, and lysosome functions (Fig. 2h–j, l). Notably, worms treated with embryo lysates showed increased fatty acid degradation gene expression and decreased fatty acid elongation gene expression (Fig. 2k, m). Given that enhanced fat degradation is linked to longer worm lifespans^34^, we used Nile Red staining to assess fat content. This revealed a significant decrease in lipid droplet intensity in embryo lysate-treated worms, consistent with their lifespan extension (Fig. 2n, o).

### A hedgehog-like signal suppresses UPR^mt^ in adult worms

**Fig. 3:**
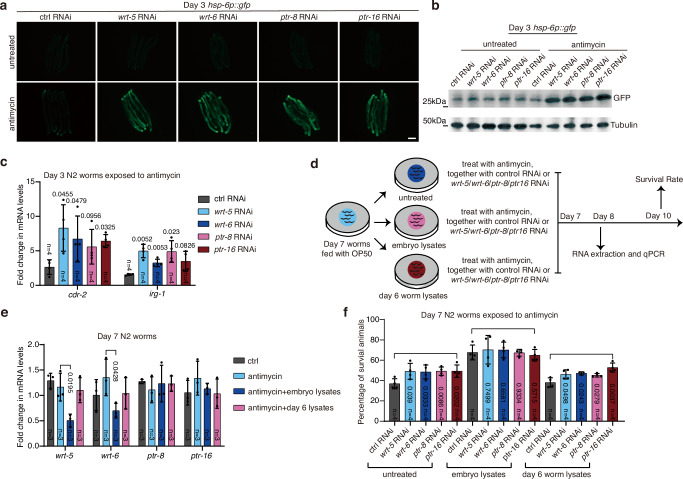
A hedgehog-like signal suppresses UPR^mt^ in adult worms. **a** Representative fluorescence images of *hsp-6p::gfp* worms with the indicated treatments. Antimycin treatment occurred on day 3 of adulthood for 24 h. Scale bar, 200 µm. **b** Immunoblotting of GFP and tubulin in lysates collected from day 3 *hsp-6p::gfp* worms with the treatments described in panel (**a**). **c** qRT-PCR analysis of the indicated UPR^mt^ genes in day 3 N2 worms subjected to the indicated treatments. **d** Schematic depicting the experimental procedure in panels (**e**, **f**). **e** qRT-PCR analysis of genes related to hedgehog signaling in day 7 N2 worms with the indicated treatments. **f** Survival rate of Day 7 N2 worms treated with embryo or worm lysates, followed by exposure to antimycin and the specified combinations of RNAi for 72 h. RNAi of individual genes started on day 7 of adulthood. Error bars indicate mean ± SD. *n* represents the number of independent experiments for panels (**c**, **e**, **f**). *p* values were assessed using a two-tailed *t* test. Source data are provided as a Source Data file.

To identify the possible germline-to-soma signals modulated by piRNAs in the embryo lysates, we knocked down genes that have been reported to mediate the communications between the germline and the soma, including genes encoding putative secreted peptides/molecules associated with the insulin pathway^35^, the hedgehog pathway^36^, the Wnt signaling pathway^37^, the prostaglandin E2 pathway^38^, Cer retrotransposons^39^ and innate immunity^40^ (Supplementary Data 2). Interestingly, knockdown of *wrt-5* and *wrt-6*, encoding hedgehog-like ligands, or *ptr-8* and *ptr-16*, encoding homologs of the hedgehog receptor Patched, directly activated UPR^mt^ in adult worms treated with antimycin, bypassing the requirement for ingestion of embryo lysates (Fig. 3a, b and Supplementary Fig. 4a–d). Accordingly, knockdown of *wrt-5*, *wrt*-*6*, *ptr-8*, or *ptr*-*16* also elevated the transcript levels of endogenous UPR^mt^ in adult worms challenged with mitochondrial insults (Fig. 3c and Supplementary Fig. 4e, f). More importantly, knockdown of other hedgehog-like family genes had no such effect (Supplementary Fig. 4g). Deficiency of *wrt-5*, *wrt-6*, *ptr-8*, or *ptr-16* did not affect the levels of UPR^mt^ activation in L4 stage larvae (Supplementary Fig. 4h, i). In adult worms, UPR^mt^ induction via knockdown of *wrt-5*, *wrt-6*, *ptr-8*, or *ptr-16* also required the transcription factor ATFS-1 and DVE-1, but not UBL-5, to elevate *hsp-6p::gfp* (Supplementary Fig. 5a, b). More interestingly, knockdown of *wrt-5*, *wrt-6*, *ptr-8*, or *ptr-16* only enabled the adult worms to regain the ability to activate UPR^mt^, but not UPR^ER^ or the heat shock response (Supplementary Fig. 5c–f). Moreover, these knockdowns did not enhance survival in adult worms subjected to ER stress or heat shock (Supplementary Fig. 5g, h). These findings suggest a specific role for hedgehog-like signaling in regulating UPR^mt^.

Ingesting embryo lysates or knockdown of *wrt-5*, *wrt-6*, *ptr-8*, or *ptr-16* enables adult worms to regain the ability to activate UPR^mt^ upon mitochondrial perturbation. Therefore, we tested if the embryo lysate treatments exert their function by inhibiting the hedgehog-like signal. Day 7 worms were fed for one day with OP50, a bacterial strain conventionally used as a food source to culture *C. elegans* in the laboratory, or with OP50 plus embryo or day 6 worm lysates. RNAs were then extracted from a sub-population of the worms to test the expression levels of *wrt-5*, *wrt-6*, *ptr-8*, and *ptr-16*. At the same time, the other sub-population of the worms was treated with antimycin, together with control RNAi, or RNAi against each of the four genes, until day 10 to measure the survival rate (Fig. 3d). Interestingly, feeding adult worms with embryo lysates reduced the transcript levels of *wrt-5* and *wrt-6*, but not *ptr-8* and *ptr-16* (Fig. 3e). Consistent with the previous report^36^, this result suggests that piRNAs in the embryo lysates transcriptionally regulate the hedgehog-like signal. However, no significant differences in the expression of *wrt-5* and *wrt-6* were observed between N2 and the piRNA pathway mutants (Supplementary Fig. 5i). We suspect that alternative mechanisms, potentially involving epigenetic regulation^41^, may compensate for the effects on *wrt-5/6* expression in these mutants. In addition, embryo lysate treatment, or RNAi of *wrt-5*, *wrt-6*, *ptr-8*, or *ptr-16*, promoted animal survival under mitochondrial stress (Fig. 3f). More importantly, the embryo lysate-induced increase in animal survival upon mitochondrial perturbation was not further elevated by single RNAi of the four genes (Fig. 3f). This indicates that the increased animal survival caused by embryo lysates ingestion was primarily through suppression of the function of the hedgehog-like signaling proteins WRT-5 and WRT-6.

**Fig. 4:**
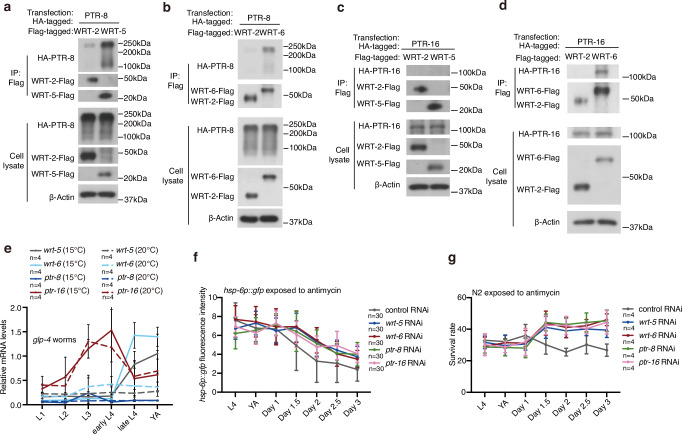
Activation of a hedgehog-like signal suppresses UPR^mt^ in adult worms. **a**-**d** Interactions of PTR-8 and PTR-16 with WRT-5 and WRT-6. HEK293T cells transfected with the indicated cDNAs and followed by the indicated immunoprecipitations. **e** qRT-PCR analysis of genes related to hedgehog signali ng in *glp-4* worms at the indicated developmental stage. Worms were raised at the specified temperatures. **f** Quantification of GFP fluorescence intensity in *hsp-6p::gfp* worms treated with antimycin. Antimycin treatment started at indicated stage for 24 h. **g** Survival rate of N2 worms after 48 h of exposure to antimycin, started at the indicated developmental stage. Error bars indicate mean ± SD. *n* represents the number of independent experiments for panels (**e**, **g**), and the number of worms for panel (**f**). Source data are provided as a Source Data file.

To explore the potential interactions between the hedgehog ligands WRT-5/6 and PTR-8/16, we conducted immunoprecipitation experiments using 293T cells overexpressing *C. elegans wrt-5/6* or *ptr-8/16*. Although the observed protein sizes for PTR-8/16 were unexpected, siRNA knockdown experiments confirmed their identities (Supplementary Fig. 5j, k). We observed interactions between WRT-5 and PTR-8, as well as between WRT-6 and both PTR-8 and PTR-16 (Fig. 4a–d). These findings suggest that PTR-8 is a potential receptor for WRT-5, while both PTR-8 and PTR-16 may serve as receptors for WRT-6.

To further investigate the precise timing of *wrt-5/6* activation, we utilized the temperature-sensitive germline mutant *glp-4*. This approach enabled us to compare relative mRNA levels with and without a functional germline, across various developmental stages. In contrast to *ptr-8* and *ptr-16*, our analysis revealed a significant increase in *wrt-5* and *wrt-6* expression in the germline during the L4 stage (Fig. 4e). Comparing to the control worms, knocking down *wrt-5*, *wrt-6*, *ptr-8*, or *ptr-16* led to enhanced activation of the UPR^mt^ and increased mitochondrial stress resistance when the worms enter adulthood (Fig. 4f, g). These results suggest that the activation of *wrt-5* and *wrt-6* is associated with the initiation of reproduction, highlighting a potential role for hedgehog-like signaling in early aging processes.

### The age-related decline in UPR^mt^ activation could be regulated by piRNAs targeting *wrt-5* and *wrt-6*

**Fig. 5:**
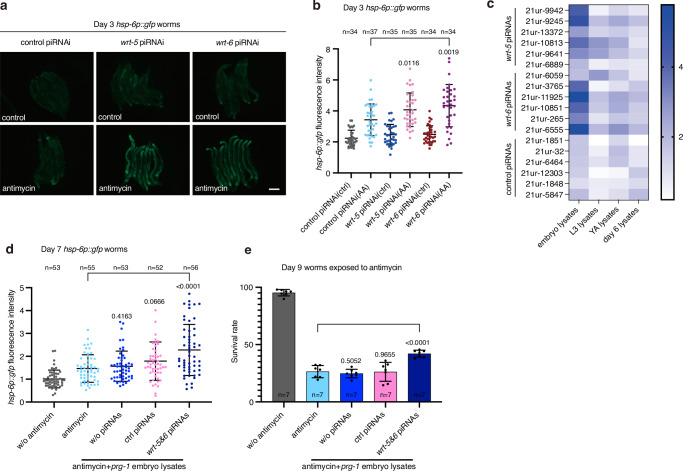
piRNAs targeting *wrt-5* and *wrt-6* block UPR^mt^ and mitochondrial stress resistance attenuation. **a** Representative fluorescence images of *hsp-6p::gfp* worms with the indicated treatments. Antimycin treatment occurred on day 3 of adulthood for 24 h. Scale bar, 200 µm. **b** Quantification of GFP fluorescence intensity in panel (**a**). **c** Heatmap showing the expression levels of piRNAs targeting *wrt-5*, *wrt-6*, and the control gene *wrt-1* in lysates from different stages. **d** Quantification of GFP fluorescence intensity in *hsp-6p::gfp* worms with the indicated treatments. Antimycin, *prg-1* embryo lysates and piRNAs were administered to day 7 worms for 24 h. **e** Survival rate of day 9 N2 worms after 72 h exposure to antimycin, *prg*-*1* embryo lysates and piRNAs. Error bars indicate mean ± SD. *n* represents the number of independent experiments for panel (**e**), and the number of worms for panels (**b**) and (**d**). *p* values were assessed using a two-tailed *t* test. Source data are provided as a Source Data file.

To determine if piRNAs are essential for regulating *wrt-5/6*, we identified six piRNAs targeting each of *wrt-5* and *wrt-6* (piRTarBase^42^; Supplementary Fig. 6a). These piRNAs were added to a synthetic piRNA clusterE^43^ (Supplementary Fig. 6a), with six piRNAs targeting *wrt-1* as controls. We found most targeted piRNAs were overexpressed, silencing *wrt-5/6* in the germline (Supplementary Fig. 6b–e). Overexpressing piRNAs against *wrt-5* or *wrt-6* activated UPR^mt^ in adult worms treated with antimycin (Fig. 5a, b and Supplementary Fig. 6f–i), but did not affect UPR^mt^ in L4 larvae (Supplementary Fig. 6j, k). Importantly, there was a noticeable decline in levels of *wrt-5/6* targeting piRNAs during germline development and aging (Fig. 5c). Furthermore, introducing synthesized *wrt-5/6*-targeting piRNAs into piRNA-devoid *prg-1* embryo lysates activated UPR^mt^ and improved survival under mitochondrial stress in lysate-fed adult worms (Fig. 5d, e). Adding *wrt-5/6*-targeting piRNAs to *prg-1* lysates from embryos, L3 stages, and young adults—but not day 6 worms—effectively promoted UPR^mt^ activation in lysate-fed adult worms (Supplementary Fig. 6l). This suggests that day 6 lysates might be missing essential components, beyond piRNAs, required to activate UPR^mt^.

### Suppression of the hedgehog-like signal increases the resistance to mitochondrial stress and pathogen infection, but reduces brood size

**Fig. 6:**
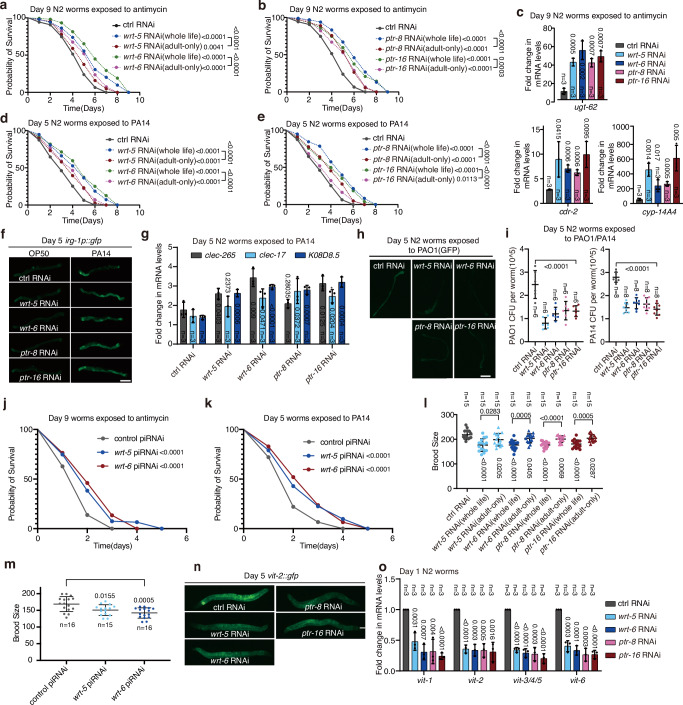
Suppression of the hedgehog-like signal increases resistance to mitochondrial stress and pathogen infection but reduces brood size. **a, b** Survival rate of day 9 wild-type (N2) worms treated with indicated RNAi and exposed to antimycin. RNAi treatment was applied either “whole-life” (starting at L1) or “adult-only” (starting on day 1 of adulthood). **c** qRT-PCR analysis of UPR^mt^ genes in day 9 N2 adults treated with RNAi and exposed to antimycin. **d**, **e** Survival rate of day 5 N2 adults treated with RNAi and exposed to PA14. **f** Representative fluorescence images of day 5 *irg-1p::gfp* adults with the indicated treatments. Pathogen infections were done on day 5 of adulthood for 48 h unless otherwise noted. Scale bar, 200 µm. **g** qRT-PCR analysis of innate immune response genes in day 5 N2 animals exposed to PA14. **h** Representative fluorescence images of day 5 N2 worms treated with PAO1(GFP). Scale bar, 200 µm. **i** Quantification of PAO1 or PA14 CFU in day 5 N2 worms. **j**, **k** Survival rates of day 9 and day 5 *hsp-6p::gfp* adults overexpressing piRNAs targeting *wrt-5*, *wrt-6*, or a control gene, with antimycin treatment on day 9 and PA14 on day 5. **l** Brood size of N2 worms treated with RNAi from L1 stage (“whole life”) or day 1 adulthood (“adult-only”). **m** Brood size of *hsp-6p::gfp* worms overexpressing piRNAs targeting *wrt-5*, *wrt-6*, or a control gene. **n** Representative fluorescence images of day 5 *vit-2::gfp* worms treated with RNAi. Scale bar, 200 µm. **o** qRT-PCR analysis of the *vit* family genes in day 1 N2 adults treated with RNAi. Error bars indicate mean ± SD. *n* represents the number of independent experiments for panels (**c**, **g**, **i**, **o**), and the number of worms for panels (**i**, **m**). *p* values were assessed using a two-tailed *t* test for panels (**c**, **g**, **i**, **l**, **m**, **o**), and using the Log-rank (Mantel–Cox) test for panels (**a**, **b**, **d**, **e**, **j**, **k**). Source data are provided as a Source Data file.

Individual knockdown of the hedgehog-like genes *wrt-5* and *wrt-6*, or the receptor genes *ptr-8* and *ptr-16*, enables the adult worms to activate UPR^mt^ under mitochondrial perturbation. Next, we tested if knockdown of these genes makes adult worms more resistant to mitochondrial stress. Indeed, knockdown of *wrt-5*, *wrt-6*, *ptr-8*, or *ptr-16* during the whole life of the worms or only in adulthood promoted the survival of adult worms exposed to mitochondrial inhibitors (Fig. 6a, b and Supplementary Fig. 7a, b). In contrast, the survival rate of L4 worms was not affected by single deficiency of these genes (Supplementary Fig. 7c, d). A prior study identified two hedgehog ligands, WRT-1 and WRT-10, which are activated by germline overactivity, resulting in worm shrinkage and mortality^36^. Unlike *wrt-5* or *wrt-6*, knocking down *wrt-1* or *wrt-10* did not activate UPR^mt^ or impact resistance to mitochondrial stress in adult worms (Supplementary Fig. 4g, 7e). Knockdown of *wrt-5*, *wrt-6*, *ptr-8*, or *ptr-16* elevated the transcript levels of stress response and innate immune response genes when the worms were exposed to mitochondrial inhibitor (Fig. 6c). Moreover, single gene deficiency also promoted the survival rate of adult worms exposed to pathogens such as *Pseudomonas aeruginosa* strains PA14 and PAO1, but not the survival of L4 worms under the same challenge (Fig. 6d, e and Supplementary Fig. 7f, g). Accordingly, lack of *wrt-5*, *wrt-6*, *ptr-8*, or *ptr-16* elevated the expression levels of innate immune response genes (Fig. 6f, g and Supplementary Fig. 7h, i) and suppressed the accumulation of pathogen in adult worms exposed to PA14 or PAO1 (Fig. 6h, i). Moreover, overexpressing piRNAs that target either *wrt-5* or *wrt-6* enhanced resistance to mitochondrial inhibitor antimycin and PA14 infection in adult worms (Fig. 6j, k). Together, these results indicate that suppressing the hedgehog-like signal enhances the resistance to mitochondrial stress and pathogen infection in adult worms.

When these four hedgehog pathway-related genes were singly knocked down starting at the L1 larval stage (during the whole life of the hatched animal), the brood size of the animals was greatly reduced (Fig. 6l). Overexpressing piRNAs that target either *wrt-5* or *wrt-6* also decreased brood size (Fig. 6m). Vitellogenin is a family of yolk proteins highly expressed in the intestine of the adult hermaphrodite and transported to the germline to support animal fertility and post-embryonic development^44^. The expression level of *vit-2*, the best-studied vitellogenin gene, was greatly reduced in worms with knockdown of *wrt-5*, *wrt-6*, *ptr-8*, or *ptr-16* (Fig. 6n, o and Supplementary Fig. 8a, b). Additionally, we assessed yolk protein (YP) levels using gel electrophoresis^45^ and revealed that knockdown of *wrt-5*, *wrt-6*, *ptr-8*, or *ptr-16* resulted in a significant reduction in YP content (Supplementary Fig. 8c–f). These data indicate that suppressing the hedgehog-like signal inhibits fertility and post-embryonic development.

### Suppressing the hedgehog-like signal extends worm lifespan

Hedgehog signaling plays a crucial and evolutionarily conserved role in development. Worms carrying single mutant alleles of hedgehog pathway-related genes *wrt-5*, *wrt-6*, *ptr-8*, and *ptr-16* exhibited developmental delays (Supplementary Fig. 8g) and commonly displayed the “exploded” phenotype during the early adult stage, due to defective vulval integrity^46^ (Supplementary Fig. 8h, i). However, worms fed with RNAi *E. coli* targeting *wrt-5*, *wrt-6*, *ptr-8*, and *ptr-16* showed no developmental delays and a lower incidence of the “exploded” phenotype at the early adult stage (Supplementary Fig. 8j, k). This may be because the RNAi partially maintains hedgehog-like signaling capacity (Supplementary Fig. 1).

**Fig. 7:**
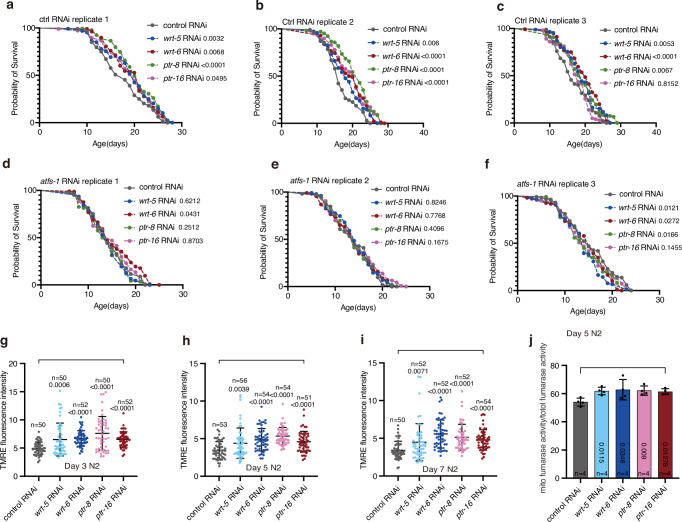
Suppressing a hedgehog-like signal extends lifespan. **a**–**f** Survival curves of control AMJ345 worms (**a**–**c**) or *atfs-1*-deficient AMJ345 worms (**d**–**f**) upon feeding with RNAi *E*. *coli* targeting a hedgehog pathway-related gene. Lifespan data from 3 biological replicates are shown. **g**-**i** Quantification of relative TMRE fluorescence in adult day 3 (**g**), day 5 (**h**), and day 7 (**i**) N2 worms. **j** Comparison of fumarase activity in different subcellular fractions in adult day 5 N2 worms. Fumarase activity was normalized to the quantity of protein used in the assay. Error bars indicate mean ± SD. *n* represents the number of independent experiments for panel j, and the number of worms for panels (**g**–**i**). *p* values were assessed using a two-tailed *t* test for panels (**g**–**j**) and the Log-rank (Mantel–Cox) test for panels (**a**–**f**). Source data are provided as a Source Data file.

Knockdown of *wrt-5*, *wrt-6*, *ptr-8*, or *ptr-16* caused approximately 30% of wild-type N2 worms to explode (Supplementary Fig. 9a). Interestingly, this phenotype occurred less frequently in germline and intestine-specific RNAi AMJ345 worms^47^ than in wild type N2 worms, suggesting that the hedgehog pathway’s role in tissues other than the germline and intestine contributes to the exploded phenotype (Supplementary Fig. 9b). Therefore, to avoid the exploded phenotype, we used AMJ345 worms for lifespan analysis. Knockdown of *wrt-5*, *wrt-6*, *ptr-8*, or *ptr-16* in germline and intestine increased lifespan (Fig. 7a–c). Additionally, deficiency of *atfs-1*, which is essential for UPR^mt^ induction, prevented the lifespan extension (Fig. 7d–f and Supplementary Fig. 9c). Accumulating evidence indicates that functional mitochondrial import machinery is crucial for UPR^mt^-mediated lifespan extension^48^. Although knockdown of *wrt-5*, *wrt-6*, *ptr-8*, or *ptr-16* did not affect the expression of mitochondrial import machinery genes (Supplementary Fig. 9d), it enhanced the mitochondrial membrane potential (ΔΨ), as indicated by increased tetramethylrhodamine ethyl ester (TMRE) staining^49^ (Fig. 7g–i). Consistently, a fumarase activity assay showed an increase in the proportion of mitochondrial to total fumarase activity following these gene knockdowns (Fig. 7j and Supplementary Fig. 9e).

### Hedgehog-like signal-regulated phenotypes are mediated via ATFS-1-dependent and independent mechanisms

Compared to N2 worms, knockdown of *wrt-5*, *wrt-6*, *ptr-8*, or *ptr-16* did not improve antimycin resistance in *atfs-1* mutants, indicating that the hedgehog-like signal promotes mitochondrial stress resistance through the transcription factor ATFS-1 (Fig. 6a, b and Supplementary Fig. 10a, b). Additionally, these knockdowns decreased survival against PA14 infection in *atfs-1* mutants relative to N2 worms (Fig. 6d, e and Supplementary Fig. 10c, d). Quantitative real-time PCR revealed that RNAi of hedgehog-like genes upregulated both ATFS-1-dependent and independent innate immunity genes^50,51^ (Supplementary Fig. 10e). The deficiency of *atfs-1* did not alleviate the reduction in brood size caused by the knockdown of hedgehog-like signaling genes (Supplementary Fig. 10f, g), demonstrating that UPR^mt^ is not essential for this reduction. These findings confirm that hedgehog-like signaling regulates innate immunity and developmental processes through ATFS-1-dependent and independent mechanisms.

### A germline-to-soma hedgehog-like signal allows worms to re-invest their resources in reproduction rather than somatic maintenance

**Fig. 8:**
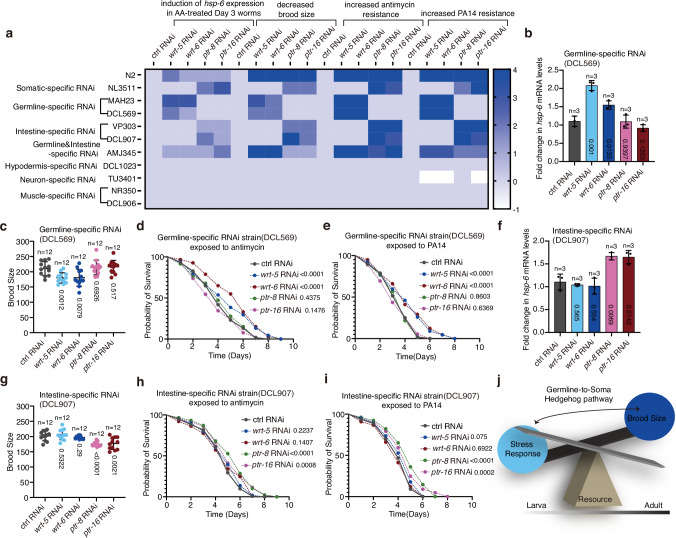
A germline-to-soma hedgehog-like signal allows adult worms to invest their resources in reproduction rather than somatic maintenance. **a** Heat map showing the effect of tissue-specific knockdown of hedgehog pathway-related genes on the indicated phenotypes. **b**, **f** qRT-PCR analysis of *hsp-6* mRNA levels in the day 3 germline- (**b**) and intestine-specific (**f**) RNAi strain treated with the indicated RNAi and antimycin. Antimycin treatment occurred on day 3 of adulthood for 24 h. **c**, **g** Brood size of the germline- (**c**) and intestine-specific (**g**) RNAi strain treated with the indicated RNAi. **d**, **h** Survival rate of the day 9 germline- (**d**) and intestine-specific (**h**) RNAi strain treated with the indicated RNAi and exposed to antimycin. **e**, **i**, Survival rate of the day 5 germline- (**e**) and intestine-specific (**i**) RNAi strain treated with the indicated RNAi and exposed to PA14. **j** A germline-to-soma hedgehog-like signal initiates resource reinvestment to promote reproduction while inhibiting somatic maintenance in adult worms. Error bars indicate mean ± SD. *n* represents the number of independent experiments for panels (**b**, **f**), and the number of worms for panels (**c**, **g**). *p* values were assessed using a two-tailed *t* test for panels (**b**, **c**, **f**, **g**), and the Log-rank (Mantel–Cox) test for panels (**d**, **e**, **h**, **i**). Source data are provided as a Source Data file.

The hedgehog-like ligands have been reported to be expressed in the germline and secreted from the germline to the soma^36^. To explore which tissues receive the hedgehog-like ligands WRT-5 and WRT-6, we employed tissue-specific RNAi strains to singly knock down the Patched receptor genes *ptr-8* and *ptr-16* in different tissues. First, we demonstrated that germline-specific single knockdown of *wrt-5* and *wrt-6*, but not *ptr-8* and *ptr-16*, allowed the adult worms to activate UPR^mt^ upon mitochondrial perturbation (Fig. 8a, b and Supplementary Fig. 11a–c). Deficiency of WRT-5 or WRT-6 in the germline also decreased the brood size and vitellogenin gene expression, and increased the resistance of animals to mitochondrial perturbations or pathogen infections (Fig. 8a, c–e and Supplementary Fig. 11d). The reduction in vitellogenin production, primarily in the intestine due to hedgehog pathway inhibition, may affect the levels of polyunsaturated fatty acids (PUFAs) in worms, and thereby affect brood size^52,53^. These results confirmed that the hedgehog-like ligands are generated in and secreted from the germline. Interestingly, single knockdown of the Patched receptor-like genes *ptr-8* and *ptr-16* in the somatic tissues or only in the intestine, but not in any other tissues such as the hypodermis, neurons, or muscles, allowed the adult worms to activate UPR^mt^ upon mitochondrial perturbation (Fig. 8a, f and Supplementary Fig. 11e–g). Deficiency of PTR-8 or PTR-16 in the intestine also reduced brood size and vitellogenin gene expression, and increased resistance to mitochondrial perturbations or pathogen infection (Fig. 8a, g–i and and Supplementary Fig. 11h and Supplementary Fig. 12a, b). These results indicate that the hedgehog-like signal is transduced from the germline to the intestine to suppress the mitochondrial stress response. When worms enter adult stage, the hedgehog-like signal allows the reproductive tissue to communicate with the key metabolic and stress response tissue to allocate limited resources for investment in reproduction, rather than maintenance of the soma (Fig. 8j).

**Fig. 9:**
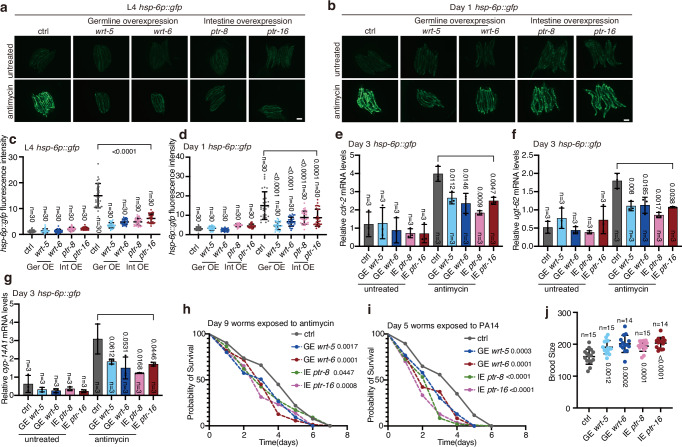
Overexpression of the hedgehog-like signal decreases resistance to mitochondrial stress and pathogen infection while increasing brood size. **a**, **b** Representative fluorescence images of *hsp-6p::gfp* worms with germline-specific *wrt-5/6* or intestine-specific *ptr-8/16* overexpression. Antimycin treatment occurred on L4 (a) or day 1 (b) of adulthood for 24 h. Scale bar, 200 µm. **c**, **d** Quantification of GFP fluorescence intensity in panels (**a**, **b**). **e**–**g** qRT-PCR analysis of the indicated UPR^mt^ genes in day 3 *hsp-6p::gfp* worms with the indicated treatments. **h**, **i** Survival rate of the day 9 (**h**) and day 5 (**i**) *hsp-6p::gfp* worms with the indicated treatments. **j** Brood size of *hsp-6p::gfp* worms with germline-specific *wrt-5/6* or intestine-specific *ptr-8/16* overexpression. Ger germline, Int intestine, OE overexpression, GE germline expression, IE intestine expression. Error bars indicate mean ± SD. *n* represents the number of independent experiments for panels (**e**–**g**), and the number of worms for panels (**c**, **d**, **j**). *p* values were assessed using a two-tailed *t* test for panels (**c**–**g**, **j**), and the Log-rank (Mantel–Cox) test for panels (**h**, **i**). Source data are provided as a Source Data file.

It has been reported that certain tissue-specific RNAi strains may also affect other tissues. For example, MAH23, commonly used for germline-specific RNAi, also partially impacts the intestine^54^ (Supplementary Fig. 12c). Therefore, we generated transgenic worms that overexpress *ptr-8/16* in the intestine or *wrt-5/6* in the germline, and observed suppressed UPR^mt^ (Fig. 9a–g). Additionally, intestine-specific overexpression of *ptr-8/16* and germline-specific overexpression of *wrt-5/6* decreased resistance to the mitochondrial inhibitor antimycin and PA14 infection, but increased brood size (Fig. 9h–j). These results confirm the specific roles of *wrt-5/6* in the germline and *ptr-8/16* in the intestine.

### The role of the hedgehog signaling pathway in mitochondrial stress response might be conserved in mammals

**Fig. 10:**
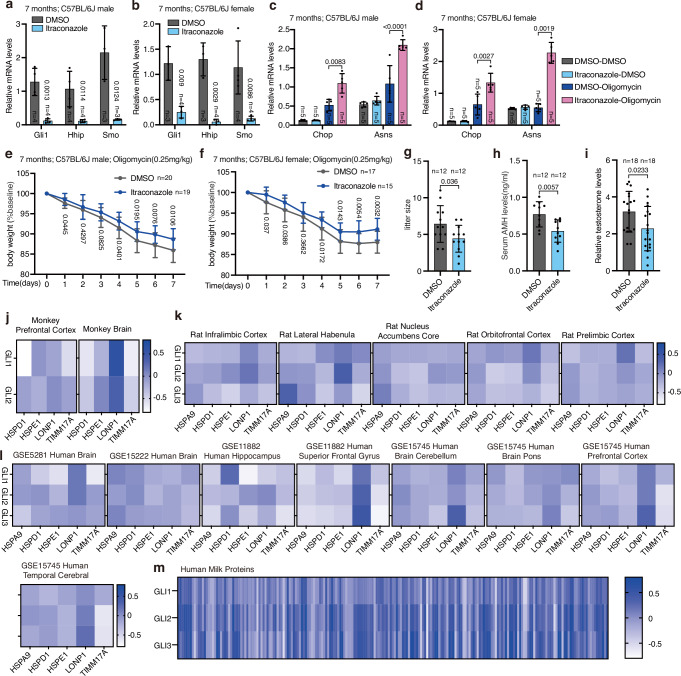
Suppression of the hedgehog-like signal increases resistance to mitochondrial stress but reduces fertility in mice. **a**, **b** qRT-PCR analysis of Gli1, Hhip, and Smo mRNA levels in seven-month-old C57BL/6J male (**a**) and female (**b**) mice treated with DMSO or itraconazole. **c**, **d** qRT-PCR analysis of Chop and Asns mRNA levels in seven-month-old C57BL/6J male (**c**) and female (**d**) mice treated with DMSO or oligomycin. **e**, **f** Weight loss in seven-month-old C57BL/6J male (**e**) and female (**f**) mice during oligomycin treatment. **g** Litter size of seven-month-old C57BL/6J females treated with DMSO or itraconazole, mated with males of the same treatment group. **h**, **i** Serum AMH (**h**) or testosterone (**i**) levels assessed by ELISA in mice treated with DMSO or itraconazole. **j**–**l** Pearson’s correlation of GLI1, GLI2, and UPR^mt^ mRNA levels in monkey (**j**), rat (**k**) and human (**l**) tissues. Blue indicates a positive correlation and white indicates a negative correlation. The intensity of the colors corresponds to the correlation coefficient. **m** Pearson’s correlation of GLI1, GLI2, GLI3, and human milk mRNA levels in human breast tissues. Blue indicates a positive correlation and white indicates a negative correlation. The intensity of the colors corresponds to the correlation coefficient. Error bars indicate mean ± SD. *n* represents the number of mice. *p* values were assessed using a two-tailed *t* test. Source data are provided as a Source Data file.

We also attempted to examine whether the role of the hedgehog signaling pathway in UPR^mt^ activation and its associated phenotypes are conserved in mammals. Mice were treated with itraconazole, an FDA-approved drug that inhibits the hedgehog signaling pathway^55^ (Supplementary Fig. 13a, b). In both male and female mice, itraconazole treatment decreased mRNA levels of Gli1, Hhip, and Smo, indicating reduced hedgehog signaling^56^ (Fig. 10a, b). Notably, suppressing the hedgehog pathway increased the transcript levels of endogenous mitochondrial stress response genes and mitigated weight loss in mice challenged with the mitochondrial inhibitor oligomycin (Fig. 10c–f and Supplementary Fig. 13c–f). Fertility tests also showed that litter sizes in seven-month-old female mice significantly decreased following hedgehog pathway inhibition (Fig. 10g). Additionally, serum anti-mullerian hormone (AMH) levels in treated female mice and testosterone levels in treated male mice were lower than in controls (Fig. 10h, i).

Mitochondria are essential organelles, particularly in the brain, one of the most energy-demanding organs. Utilizing the GeneNetwork database, we found negative correlations between mRNA levels of hedgehog pathway zinc finger proteins GLIs and UPR^mt^-related transcripts (HSPA9, HSPD1, HSPE1, LONP1, and TIMM17A) in various rat, monkey, and human brain tissues (Fig. 10j–l). Additionally, mRNA levels of GLI1, GLI2, and GLI3 negatively correlated with these UPR^mt^ transcripts in multiple human tissues including adipose, esophageal, arterial, pulmonary, muscular, and hepatic tissues (Supplementary Fig. 13g). Conversely, in human breast tissue, GLI1, GLI2, and GLI3 expression positively correlated with transcripts of human milk genes (Fig. 10m). These findings indicate that the hedgehog signaling pathway may play a conserved role in regulating UPR^mt^ and fertility across species.

## Discussion

The ability to cope with different cellular stresses declines during animal aging. However, the underlying mechanisms remain elusive. Using *C. elegans* as a model organism, we found that a hedgehog-like signal, produced in the germline, suppressed the mitochondrial stress response in the intestine in adult animals. More importantly, this study uncovers the biological meaning of the age-related decline of mitochondrial stress response from an evolutionary point of view.

Several hypotheses have been proposed to explain why biological aging occurs. The “antagonistic pleiotropy” (multiple effects of a single gene) theory postulates that genes beneficial for early life are strongly selected but can be detrimental in later life when selection wanes after reproduction^57^. The “disposable soma” theory postulates that aging reflects the decision to allocate limited resources for investment in early life reproduction rather than maintenance of the soma to extend the lifespan^58^. The current findings support the “antagonistic pleiotropy” and the “disposable soma” theories. The genes related to hedgehog signaling are beneficial at the larval stages when they promote development and reproduction, but are detrimental in later life when they suppress the mitochondrial stress response. Moreover, the generation and transmission of the hedgehog-like signal from the germline to the soma suppresses the mitochondrial stress response in the somatic tissues, allowing the worms to invest more resources in reproduction rather than somatic maintenance.

The initiation of this project was stemming from an artificial experiment where we fed adult *C. elegans* with embryo lysates and noted the induction of UPR^mt^. This approach was instrumental in identifying potential factors capable of modulating UPR^mt^ in adults. Subsequent investigations revealed that germline-expressed piRNAs played a significant role, with their levels markedly decreasing in young adults. This reduction may facilitate the production and secretion of a Hedgehog-like signal that suppresses somatic UPR^mt^. While the initial experiment with embryo lysates was artificial, it paved the way for discovering a naturally occurring germline-to-soma communication mechanism. Our findings demonstrate that the signal is primarily transmitted from the germline, underscoring its physiological relevance.

Our findings illustrate the role of *wrt-5/*6-tageting piRNAs in regulating UPR^mt^ across various developmental stages of *C. elegans*. Notably, our analysis indicates a sustained expression of certain *wrt-5/6*-targeting piRNAs into the L3 and young adult phases (Fig. 5c), albeit at lower levels than observed in embryos, which appears to contribute to UPR^mt^ activation. This is further corroborated by our experiments showing that UPR^mt^ could not be activated in adult worms using L3 extracts from *prg-1* mutants, underscoring the critical function of piRNAs in this context. piRNAs are thought to function by generating 22G-RNAs. We conducted small RNA sequencing on worms treated with embryo lysates but found no significant changes in 22G-RNAs following either embryo lysate feeding or antimycin treatment. This lack of significant changes may be due to the timing of the sequencing, performed 24 h post-treatment, which corresponds with UPR^mt^ activation but may miss earlier events necessary for 22G-RNAs production. As a result, there is currently no strong evidence supporting 22G-RNAs in regulating *wrt-5/6* transcripts. Further studies are required to uncover the exact mechanisms by which piRNAs regulate this process. Moreover, our exploration extends to identifying additional factors within the lysates from embryos, L3, and young adult stages that might enhance piRNA-mediated UPR^mt^ induction. This premise gains support from our observations where the mere addition of piRNAs to day 6 *prg-1* lysates was insufficient to trigger UPR^mt^ (Supplementary Fig. 6l), suggesting the absence of other essential components in day 6 lysates that are pivotal for UPR^mt^ induction. These insights collectively highlight the complex interplay between piRNAs and other molecular constituents in modulating the mitochondrial stress response, suggesting a broader regulatory landscape that extends beyond piRNA action alone.

Previous research demonstrated that PGL-1 aggregation in the germline, induced by the loss of CEY factors (Y-box binding proteins), affects not only the mitochondrial network in the germline but also triggers UPR^mt^ activation in somatic tissues via Wnt signaling^37^. During the L4 and YA stages, mitochondrial DNA (mtDNA) copy number significantly increases in the germline^59^. Our findings suggest the L4 stage as critical for activating germline hedgehog-like signaling. Future research could explore how this surge in mitochondrial activity and protein synthesis during the L4 and YA stages influences hedgehog-like signaling and its coordination with the piRNA pathway for resource allocation between reproduction and soma maintenance. While this manuscript was being prepared, it was reported that the germline functions as a central signaling hub, regulating neuron-to-intestine cell-nonautonomous UPR^mt^ activation, emphasizing the important role of the germline in maintaining mitochondrial health^60^.

Our study underscores the germline-to-soma signaling pathway’s role in modulating the UPR^mt^, yet it’s important to acknowledge that germline signals can also affect other proteostatic responses, such as the heat shock response (HSR)^61,62^. Whether UPR^ER^ is regulated in a similar manner requires further exploration. The selective regulatory mechanisms by which germline signals influence specific somatic stress responses are yet to be fully understood. Delving into the mechanisms through which germline signals selectively modulate distinct somatic stress pathways could offer profound insights into how organismal stress responses are coordinated, and the intricate dynamics between reproductive status and somatic health.

## Methods

### *C. elegans* strains and culture

Worms were grown and maintained at 20 °C and fed with *E. coli* OP50 on Nematode Growth Medium (NGM) (0.25% Bacto peptone, 0.3% sodium chloride, 1.7% agar, 5 μg/mL cholesterol, 25 mM potassium phosphate pH 6.0, 1 mM magnesium sulfate, 1 mM calcium chloride) unless otherwise indicated. Strains used in this study are detailed in Supplementary Data 3.

### Bacterial strains and culture

Bacterial strains used in this study were *Escherichia coli* strains OP50 and HT115, and *Pseudomonas aeruginosa* strains PA14, PAO1 and PAO1(GFP). OP50 was cultured with LB medium at 37 °C unless otherwise indicated. HT115, PA14, PAO1 and PAO1(GFP) were cultured with LB medium containing ampicillin (50 mg/ml) at 37 °C unless otherwise indicated.

### Induction of UPR^mt^

For induction of UPR^mt^ in worms by treatment with mitochondrial inhibitor, unless otherwise stated, synchronized L1 worms were raised on NGM plates at 20 °C. Adult worms were transferred every day to new plates. Worms were transferred to NGM plates containing mitochondrial inhibitor (1 μg/ml antimycin, 20 μM DECA, 20 μM CCCP, 0.5 mM paraquat) at the indicated time stage. After 24 h, worms were imaged.

For induction of UPR^mt^ by feeding worms with PA14, synchronized L1 worms were raised on NGM plates at 20 °C. Adult worms were transferred every day to new plates starting at day 3 of adulthood. Worms were transferred to PA14 slow-killing NGM plates. After 48 h, worms were imaged.

For the induction of UPR^mt^ in worms by RNAi, synchronized L1 worms were cultured on NGM plates at 20 °C. Adult worms were transferred to new plates daily starting on day 3 of adulthood. The worms were synchronized and raised on NGM plates containing *atp-2*, *cco-1*, or *spg-7* RNAi. After 48 h, the worms were imaged.

### Induction of UPR^ER^

Synchronized L1 worms were raised on NGM plates at 20 °C. Adult worms were transferred every day to new plates. Worms were transferred to NGM plates containing tunicamycin (4 μg/ml) at the indicated stage. After 24 h, GFP fluorescence intensity was quantified.

### Induction of heat shock response

Synchronized L1 worms were raised on NGM plates at 20 °C. Adult worms were transferred every day to new plates. Worms were transferred to pre-heated NGM plates at the indicated stage, cultured at 33 °C for 2 h and then transferred to 20 °C. After 24 h, GFP fluorescence intensity was quantified.

### Induction of innate immune response

Synchronized L1 worms were raised on NGM plates at 20 °C. Adult worms were transferred every day to new plates. At day 5 of adulthood, worms were transferred to PA14 slow killing plates. Wild-type worms were collected for RNA extraction after 48 h. *irg-1p::gfp* worms were imaged after 48 h.

### Drug killing assay and *Pseudomonas aeruginosa* slow killing assay

Synchronized L1 worms were raised on NGM plates at 20 °C. Adult worms were transferred every day to new plates. 50-100 worms were transferred randomly to NGM plates containing different drugs (2 μg/ml antimycin, 30 μM DECA, 30 μM CCCP, 1 mM paraquat, 4 μg/ml tunicamycin) at the indicated stage. For Fig. 3m, worms were treated with 4 μg/ml antimycin. Living worms were counted every day, until all worms were dead. Animals that crawled off the plate or died due to vulva bursting were excluded. During the experiments, worms were transferred to new plates containing drug at the appropriate time if viable offspring were produced. To measure 72-h survival rates, the total number of worms was counted at the beginning of the experiment, and the number of surviving worms was counted 72 h later. For Fig. 3m, we measured 48-h survival rates.

*Pseudomonas aeruginosa* (PA) was cultured in LB containing 50 mg/ml ampicillin at 37 °C for 16 h. Concentrated PA were seeded onto air-dried slow killing plates, then incubated at 37 °C for 24 h and subsequently at room temperature for another 24 h. Synchronized L1 worms were raised on NGM plates at 20 °C. Adult worms were transferred every day to new plates. 50-100 worms were transferred to slow killing plates at the indicated stage. Living worms were counted every day until all worms were dead. Animals that crawled off the plate or died due to vulva bursting were excluded. During the experiments, worms were transferred to new plates containing drug at the appropriate time if viable offspring were produced.

The selection of Days 5 and 9 for the antimycin and PA14 survival experiments was aimed at identifying the most suitable time points for plotting survival curves. This choice helps avoid scenarios where median survival times are either too long or too short, which can obscure discernible differences.

### Thermo-tolerance assay

Synchronized L1 worms were raised on NGM plates at 20 °C. Adult worms were transferred every day to new plates. The worms were transferred to pre-heated NGM plates at the indicated stage, cultured at 37 °C for 4 h and then transferred to 20 °C. After 72 h, living worms were counted.

### Lifespan

For the lysate-treated lifespan assays, synchronized L1 worms were cultured on NGM plates at 20 °C. Starting from Day 1, worms were transferred daily to new plates. From Day 3 to Day 7, they were moved daily to fresh lysate-treated NGM plates, and then every other day to fresh NGM plates from Day 7 onwards. For the RNAi-treated lifespan assays, synchronized L1 worms were fed either control RNAi *E. coli* or RNAi *E. coli* targeting hedgehog-related genes. From Day 1 to Day 7, worms were transferred daily to fresh RNAi plates, and then every other day from Day 7 onwards. For all experiments, living worms were counted daily until all had died. Animals that crawled off the plates or died due to vulva bursting were censored. No 5-fluoro-2′-deoxyuridine (FUDR) was added to the plates.

### Preparation of embryo/worm lysates

Day 1 hermaphrodites were collected from plates and washed 3 times in M9 buffer (42.3 mM Na_2_HPO_4_, 22.1 mM KH_2_PO_4_, 85.5 mM NaCl). The worms were then bleached to release the embryos at mixed developmental stages. Day 6 hermaphrodites were collected from plates and washed 3 times in M9 buffer. The embryo or worm pellet were washed and resuspended with PBS. Dounce tissue grinders were used to homogenize embryos or worms. A dissection microscope was used to monitor the level of homogenization to ensure no visible intact worms or embryos in the lysates. The homogenizing process was carried out on ice. Each lysate within an experiment was normalized to the starting volume of embryos/worm pellet. After homogenizing, lysates were cleared by centrifugation at 3000 *g* at 4 °C for 1 min. For treatment of the lysates with different enzymes, Proteinase K (0.1 mg per lysate sample derived from 2 million embryos), RNase AT1 (0.1 mg per lysate sample derived from 2 million embryos), or DNase I (0.1 mg per lysate sample derived from 2 million embryos) were added to the lysates. The lysates were then incubated at 37 °C and gently rotated at 500 rpm for 1 h. To feed worms with lysate, 300 µl of embryo lysate (derived from 2 million embryos) or 300 ul of worm lysate (derived from 100,000 worms) was immediately spread onto 3.5 cm NGM OP50 plates. Synchronized worms at the indicated stage were then transferred to the lysate-treated plates.

### Germline dissection

For germline dissection, synchronized worms were placed in M9 buffer containing 2 mM levamisole. Using two syringe needles, worms were decapitated with a single scissoring motion, allowing one arm of the gonad to be fully released from the body cavity. The gut, appearing darker and uniform in width, contrasts with the clear gonad which has a distal tapered tip. Immediately after dissection, the germlines were placed into tubes containing 500 µl of TransZol Up reagent (TransGen Biotech, #ET111-01). Approximately 50 germlines were collected for each biological replicate.

### Germline piRNA overexpression

For germline piRNA overexpression, the piRTarBase (http://cosbi6.ee.ncku.edu.tw/piRTarBase/) was utilized to predict piRNAs related to *wrt-5* and *wrt-6*. The piRNA-mediated interference system (‘piRNAi’) was employed to overexpress endogenous piRNAs. Scaffold information for the piRNA expression cluster E was obtained from https://www.wormbuilder.org/piRNAi/. Overexpression clusters targeting *wrt-5* and *wrt-6*, as well as a control piRNA overexpression cluster, were synthesized by Xianghong Biotech. Transgenic animals harboring piRNAi transgenes were created by Suny Biotech following standard protocols. The injection mix for creating transgenes included 10 ng/µl of synthetic dsDNA for piRNA overexpression cluster and 10 ng/µl of a co-injection marker (*myo-2p::mCherry*). The sequences of the piRNA overexpression clusters used in this study are listed below:

piRNA overexpression cluster targeting *wrt-5* (uppercase: piRNAs)

cgcgcttgacgcgctagtcaactaacataaaaaaggtgaaacattgcgaggatacatagaaaaaacaatacttcgaattcatttttcaattacaaatcctgaaatgtttcactgtgttcctataagaaaacattgaaacaaaatattaagtTAATTTCTTCAATTTCTTCAActaattttgattttgattttgaaatcgaatttgcaaatccaattaaaaatcattttctgataattagacagttccttatcgttaattttattatatctatcgagttagaaattgcaacgaagataatgtcttccaaatactgaaaatttgaaaatatgttTTTGGCTCAAAATCTGAAAAAattgccagaactcaaaatatgaaatttttatagttttgttgaaacagtaagaaaatcttgtaattactgtaaactgtttgctttttttaaagtcaacctacttcaaatctacttcaaaaattataatgtttcaaattacataactgtgtGTAACGTGAAAAAATTGAGGAactgtagagcttcaatgttgataagatttattaacacagtgaaacaggtaatagttgtttgttgcaaaatcggaaatctctacatttcatatggtttttaattacaggtttgttttataaaataattgtgtgatggatattattttcagacctcatactaatctgcaaaccttcaaacaatatgtgaagtctactctgtttcactcaaccattcatttcaatttggaaaaaaatcaaagaaatgttgaaaaattttcctgtttcaacattatgacaaaaatgttatgattttaataaaaacaatTAAATATAAAAAAACGGAATAttctgtttttcttagaagtgttttccggaaacgcgtaattggttttatcacaaatcgaaaacaaacaaaaatttttttaattatttctttgctagttttgtagttgaaaattcactataatcatgaataagtgagctgcccaagtaaacaaagaaaatttggcagcggccgacaactaccgggttgcccgatttatcagtggaggaCTTTTTTCTTTTTATTTTTCAatctaatgtgatgtacacggttttcatttaaaaacaaattgaaacagaaatgactacattttcaaattgtctatttttgctgtgtttattttgccaccaacaatTTAGGAAGACATAAATAATTGtcaatctagtaaactcacttaatgcaattcctccagccacatatgtaaacgttgtatacatgcagaaaacggttttttggttttaatgggaacttttgacaaattgttcgaaaatcttaagctgtcccatttcagttgggtgatcgattt.

piRNA overexpression cluster targeting *wrt-6* (uppercase: piRNAs)

cgcgcttgacgcgctagtcaactaacataaaaaaggtgaaacattgcgaggatacatagaaaaaacaatacttcgaattcatttttcaattacaaatcctgaaatgtttcactgtgttcctataagaaaacattgaaacaaaatattaagtTGTGAGAAGGGAATTTGTCGActaattttgattttgattttgaaatcgaatttgcaaatccaattaaaaatcattttctgataattagacagttccttatcgttaattttattatatctatcgagttagaaattgcaacgaagataatgtcttccaaatactgaaaatttgaaaatatgttCTCGGAAAATAAAATAATTAAattgccagaactcaaaatatgaaatttttatagttttgttgaaacagtaagaaaatcttgtaattactgtaaactgtttgctttttttaaagtcaacctacttcaaatctacttcaaaaattataatgtttcaaattacataactgtgtGAAAATCTGATAACTATGAAAactgtagagcttcaatgttgataagatttattaacacagtgaaacaggtaatagttgtttgttgcaaaatcggaaatctctacatttcatatggtttttaattacaggtttgttttataaaataattgtgtgatggatattattttcagacctcatactaatctgcaaaccttcaaacaatatgtgaagtctactctgtttcactcaaccattcatttcaatttggaaaaaaatcaaagaaatgttgaaaaattttcctgtttcaacattatgacaaaaatgttatgattttaataaaaacaatTTTTGTATTCGTATTCATTGTttctgtttttcttagaagtgttttccggaaacgcgtaattggttttatcacaaatcgaaaacaaacaaaaatttttttaattatttctttgctagttttgtagttgaaaattcactataatcatgaataagtgagctgcccaagtaaacaaagaaaatttggcagcggccgacaactaccgggttgcccgatttatcagtggaggaAAAATAAAGAATTCCATTCAAatctaatgtgatgtacacggttttcatttaaaaacaaattgaaacagaaatgactacattttcaaattgtctatttttgctgtgtttattttgccaccaacaatTATAATCGTGGTATTTGCTCGtcaatctagtaaactcacttaatgcaattcctccagccacatatgtaaacgttgtatacatgcagaaaacggttttttggttttaatgggaacttttgacaaattgttcgaaaatcttaagctgtcccatttcagttgggtgatcgattt.

piRNA overexpression cluster control (uppercase: piRNAs)

cgcgcttgacgcgctagtcaactaacataaaaaaggtgaaacattgcgaggatacatagaaaaaacaatacttcgaattcatttttcaattacaaatcctgaaatgtttcactgtgttcctataagaaaacattgaaacaaaatattaagtTTTATTGGGTATTGGAATTGActaattttgattttgattttgaaatcgaatttgcaaatccaattaaaaatcattttctgataattagacagttccttatcgttaattttattatatctatcgagttagaaattgcaacgaagataatgtcttccaaatactgaaaatttgaaaatatgttCCTTTTATAAAGCTGAAAATAattgccagaactcaaaatatgaaatttttatagttttgttgaaacagtaagaaaatcttgtaattactgtaaactgtttgctttttttaaagtcaacctacttcaaatctacttcaaaaattataatgtttcaaattacataactgtgtGGTCCTGAAAATAAAGAAAGAactgtagagcttcaatgttgataagatttattaacacagtgaaacaggtaatagttgtttgttgcaaaatcggaaatctctacatttcatatggtttttaattacaggtttgttttataaaataattgtgtgatggatattattttcagacctcatactaatctgcaaaccttcaaacaatatgtgaagtctactctgtttcactcaaccattcatttcaatttggaaaaaaatcaaagaaatgttgaaaaattttcctgtttcaacattatgacaaaaatgttatgattttaataaaaacaatTTCCATCACTCAGAAATCTGTttctgtttttcttagaagtgttttccggaaacgcgtaattggttttatcacaaatcgaaaacaaacaaaaatttttttaattatttctttgctagttttgtagttgaaaattcactataatcatgaataagtgagctgcccaagtaaacaaagaaaatttggcagcggccgacaactaccgggttgcccgatttatcagtggaggaTGTTCTAAAGATGAAGTTCTAatctaatgtgatgtacacggttttcatttaaaaacaaattgaaacagaaatgactacattttcaaattgtctatttttgctgtgtttattttgccaccaacaatTTTATTTTTCCTACGTTTTAAtcaatctagtaaactcacttaatgcaattcctccagccacatatgtaaacgttgtatacatgcagaaaacggttttttggttttaatgggaacttttgacaaattgttcgaaaatcttaagctgtcccatttcagttgggtgatcgattt.

### piRNA feeding

For the piRNA feeding assay, piRNAs targeting *wrt-5*, *wrt-6*, and a control gene *wrt-1* were synthesized by Xianghong Biotech. 60 µg of total piRNAs (10 µg per single piRNA) were combined with *prg-1* embryo or worm lysates and then applied directly to OP50-seeded NGM plates. Synchronized worms at the indicated stage were subsequently transferred to these lysate-seeded plates. The piRNA oligos were 5′-monophosphorylated and 2′-O-methylation at their 3′ termini. The piRNA oligos used were:

21ur-9942: 3′ AACUUCUUUAACUUCUUUAAU 5′

21ur-9245: 3′ AAACCGAGUUUUAGACUUUUU 5′

21ur-13372: 3′ CAUUGCACUUUUUUAACUCCU 5′

21ur-10813: 3′ AUAAGGCAAAAAAAUAUAAAU 5′

21ur-9641: 3′ GAAAAAAGAAAAAUAAAAAGU 5′

21ur-6889: 3′ GUUAAUAAAUACAGAAGGAUU 5′

21ur-6059: 3′ AGCUGUUUAAGGGAAGAGUGU 5′

21ur-3765: 3′ GAGCCUUUUAUUUUAUUAAUU 5′

21ur-11925: 3′ CUUUUAGACUAUUGAUACUUU 5′

21ur-10851: 3′ UGUUACUUAUGCUUAUGUUUU 5′

21ur-265: 3′ UUUUAUUUCUUAAGGUAAGUU 5′

21ur-6555: 3′ GCUCGUUUAUGGUGCUAAUAU 5′

21ur-5847: 3′ AGUUAAGGUUAUGGGUUAUUU 5′

21ur-4659: 3′ GGAAAAUAUUUCGACUUUUAU 5′

21ur-12303: 3′ CCAGGACUUUUAUUUCUUUCU 5′

21ur-6464: 3′ UGUCUAAAGACUCACUACCUU 5′

21ur-32: 3′ ACAAGAUUUCUACUUCAAGAU 5′

21ur-1851: 3’ AAUUUUGCAUCCUUUUUAUUU 5′

### Nile Red staining

For the Nile Red staining assay, Nile Red was added into OP50-seeded NGM plates to achieve a final concentration of 0.05 µg/ml in the agar. Synchronized worms were cultured on OP50-seeded NGM plates starting from the L1 stage. By day 7, the worms were transferred to OP50-seeded NGM plates containing Nile Red. After 48 h, the worms were imaged using a Zeiss Imager M2 microscope and the images were analyzed with ImageJ software.

### TMRE staining

For the tetramethylrhodamine ethyl ester (TMRE) staining assay, TMRE was added to OP50-seeded NGM plates at a final concentration of 0.1 µM. Synchronized worms were cultured on RNAi plates starting from the L1 stage. At day 1, 3, or 5, the worms were transferred to RNAi plates that contained TMRE. After 48 h, the worms were imaged using a Zeiss Imager M2 microscope and the images were analyzed using ImageJ software.

### Fumarase assay

Synchronized worms were harvested from the plates and washed at least three times with M9 buffer. The worms were then homogenized using Dounce tissue grinders. Cytosolic and mitochondrial fractions were separated via differential centrifugation using a Fumarate Hydratase Assay Kit (Grace Biotech, G0869F). Protein concentrations in each fraction were quantified using a BCA assay (Thermo Scientific, 23225). Fumarase activity in these fractions was measured with the Fumarate Hydratase Assay Kit.

### Microscopy

Synchronized worms were randomly picked into 2 mM levamisole droplets on 2% agarose pads and imaged by a Zeiss Imager M2 microscope. Exposure time was the same in each experiment. At least three biologically independent experiments were performed and similar results were obtained.

### RNA isolation and real-time PCR

Synchronized worms were collected from the plates, washed at least three times with M9 buffer, and resuspended in TransZol Up reagent (TransGen Biotech, #ET111-01). Worm samples were frozen in liquid nitrogen and freeze-thawed for at least five times. Total RNA was isolated by chloroform extraction followed by isopropanol precipitation, washed with 75% (v/v) ethanol and dissolved in RNase-free water. cDNA was then synthesized using EasyScript® One-Step gDNA Removal and cDNA Synthesis SuperMix (TransGen Biotech, #AE311-02) kits. Quantitative PCR was carried out using a SYBR® Green Premix Pro Taq HS qPCR Kit (Accurate Biology #AG11701). For quantification, mRNA levels were normalized to *rpl32*. For piRNA detection, cDNA was synthesized using a miRNA 1st Strand cDNA Synthesis Kit (Vazyme Biotech, MR101-01). PCR amplification was performed with a piRNA-specific forward primer and a universal reverse primer. Quantification of piRNA levels was normalized to U6. Supplementary Data 4 summarizes the primer sequences for quantitative RT-PCR.

### Western blotting

Synchronized worms were collected from plates, washed at least three times with M9 buffer, and then resuspended with SDS loading buffer (100 mM Tris-HCl pH 6.8, 4% SDS, 20% glycerol, 10% β-mercaptoethanol, 0.004% bromophenol blue) and boiled at 100 °C for 15 min. Lysates containing the same amount of protein were loaded into an SDS-PAGE gel, followed by electrophoresis and transfer. 5% non-fat milk was used to block PVDF membranes. The membranes were then incubated with the designated primary and secondary antibodies. In this study, the following primary antibodies were used: anti-GFP (Sungene #KM8009, 1:2000 dilution), anti-tubulin (Abcam #ab6161, 1:1000 dilution), anti-Flag (Sigma #F7425, 1:2000 dilution), anti-HA (CST #3724, 1:2000 dilution), anti-b-actin (ABclonal #AC026, 1:1000 dilution), anti-HSP-60 (CST #4870, 1:1000 dilution). The membranes were developed with the enhanced chemiluminescence method (Thermo) and visualized by a Tanon 5200 chemical luminescence imaging system.

### RNA interference

Some RNAi clones were obtained from the Ahringer library; other RNAi clones were generated by PCR amplification of worm cDNA or gDNA and ligated into L4440 vector. Supplementary Data 5 summarizes the information about all RNAi clones. After sequencing verification, RNAi plasmids were transformed into HT115 competent cells (ZOMANBIO, #ZC1233). HT115 clones were cultured in LB containing 50 mg/ml ampicillin at 37 °C for 12 h. IPTG was then added to the final concentration of 1 mM. After 12 h, *E. coli* was concentrated and then seeded onto a plate containing 1.2 mg/ml IPTG. The plates were air-dried and incubated at 37 °C overnight. Worms were transferred onto the plates when the plate temperature dropped to 20 °C. For double RNAi treatment, the concentrated RNAi clones were mixed at a 1:1 ratio and seeded on the plates. All RNAi treatments were started upon hatching and proceeded throughout post-embryonic development and adulthood, unless otherwise indicated. Control RNAi experiments were carried out using an empty vector plasmid L4440.

### RNA screening

RNAi screening was performed by feeding worms with *Escherichia coli* HT115. RNAi clones were taken from the Ahringer library. Embryos from *hsp-6p::gfp* worms were collected by bleach synchronization, then 80-100 synchronized L1 worms were added to each well of 12-well plates containing RNAi bacteria. Control RNAi experiments were carried out using an empty vector plasmid L4440. Adult worms were transferred every day to new wells containing RNAi bacteria. Larval L4 or adult day 3 worms were transferred to RNAi NGM plates containing mitochondrial inhibitor (1 µg/ml antimycin). After 24 h, worms were scored. Scores were recorded from ns (no induction of GFP expression) to +++ (strong induction of GFP expression).

### Quantification of PAO1(GFP) and PA14 colony-forming units (CFU)

Synchronized worms at the indicated stage were exposed to PAO1(GFP) or PA14. After 48 h, worms were washed at least three times with M9 buffer, then transferred to M9 buffer containing 1 mg/ml kanamycin and rotated for 20 min. Washing and kanamycin treatment were repeated for at least four times to kill external bacteria on the surface of worms. Worms were then spread on an empty plate and air-dried. 20 worms were randomly picked into 100 μl M9 buffer and ground with a motorized pestle. Worm lysates were serially diluted. For each dilution, triplicates of 10 μl suspension were dropped onto LB plates containing 50 mg/ml of ampicillin. After overnight cultivation at 37 °C, the colonies were counted

### Brood size

Synchronized L4 worms were transferred to RNAi plates (one worm per plate). Worms were transferred to new RNAi plates every day until day 3 of adulthood, and then transferred to new RNAi plates every two days, until reproduction ceased. Offspring were allowed to grow at 20 °C for 24-48 h and then counted. Offspring from 10–20 individual worms were counted for each condition.

### Larval developmental assay

To quantify developmental delay caused by knocking down hedgehog-related genes, synchronized L1 wild-type worms were fed with control RNAi *E.coli* or RNAi *E.coli* targeting hedgehog-related genes. To quantify developmental delays caused by knocking out hedgehog-related genes, wild-type worms and hedgehog-related mutants were fed with OP50 starting at the L1 stage. Worms were cultured at 20 °C for 55 h. The developmental stages of worms were analyzed. Larval stages were visually determined based on vulval development.

### Exploded phenotype assay

To quantify the exploded (ruptured vulva) phenotype resulting from the knockdown of hedgehog-related genes, synchronized L1 wild-type worms were fed with control RNAi *E. coli* or RNAi *E. coli* targeting hedgehog-related genes. To assess the exploded phenotype upon knockout of hedgehog-related genes, wild-type worms and hedgehog-related gene mutants were fed with OP50 starting at the L1 stage. Data on the exploded phenotype were collected from day 1 to day 5 of adulthood. For the lifespan assay, data on the exploded phenotype were collected throughout all life stages.

### PAO1(GFP) intestinal accumulation assay

PAO1(GFP) was cultured in LB containing 50 mg/ml ampicillin at 37 °C for 16 h. Concentrated PAO1(GFP) were seeded onto slow-killing plates, air-dried and incubated at 37 °C for 24 h and then at room temperature for another 24 h. Synchronized L1 worms were raised on control or hedgehog-related gene RNAi plates at 20 °C. Adult worms were transferred every day to new plates. At day 5 of adulthood, worms were transferred to PAO1(GFP) plates. After 48 h, worms were washed at least three times with M9 buffer and imaged.

### *vit-2::gfp* observation assay

Synchronized L1 worms were raised on control or hedgehog-related gene RNAi plates at 20 °C. Adult worms were transferred every day to new plates. *vit-2::gfp* images were taken at day 5 of adulthood. Fluorescence intensity was quantified using ImageJ.

### Yolk protein proportion measurements

For yolk protein proportion measurements, 100 day 1 N2 worms were picked into M9 buffer and washed at least three times with M9 buffer. Subsequently, 100 µL of 2x Laemmli sample buffer (Sigma) was added. The samples were incubated at 95 °C, vortexed continuously for 10 min, and then loaded onto Criterion XT precast gels (4%–12% Bis-Tris, Bio-Rad) using XT MOPS (Bio-Rad) as the running buffer. Gels were stained and destained following standard Coomassie blue dye protocols. Yolk proteins (YP170, YP115, and YP88) were identified based on published data and normalized to myosin.

### mRNA-seq sample preparation and data analysis

To prepare the mRNA-seq samples, approximately 500 synchronized day 7 N2 worms were treated with either untreated or treated with embryo lysates for 24 h. After treatment, the worms were washed, collected with M9 buffer, and resuspended in TransZol Up reagent (TransGen Biotech, #ET111-01). Total RNA was then isolated using chloroform extraction followed by isopropanol precipitation. Sequencing was conducted by BGI Tech, with sequencing quality evaluation performed using FastQC (version 0.11.9) prior to mapping.

The reads were aligned to the *Caenorhabditis elegans* genome (Ensembl WBcel235/ce11 assembly) using HISAT2 (version 2.2.1) with default parameters, retaining only uniquely mapped reads for further analysis. Gene annotations for WBcel235/ce11 in GTF format were sourced from Ensembl (ftp://ftp.ensembl.org/pub/release-110/gtf/caenorhabditis_elegans/). Gene count matrices were generated using HTSeq-count from HTSeq (version 0.12.4).

Differential expression analysis was performed using DESeq2 (version 1.26.0). Genes with low expression (FPKM < 0.5 in all samples) were excluded from the analysis. Genes with an adjusted *p*-value below 0.05 were considered significantly differentially expressed. Finally, KEGG enrichment analysis of the differentially expressed genes was performed using the clusterProfiler R package (version 4.10.0). The hypergeometric distribution test is used to determine whether a known biological function or process is enriched for the differentially expressed genes. All expressed genes are taken as the total number of backgrounds N, the total number of genes annotated to a certain subset of known gene sets (KEGG) is M, the number of differentially expressed genes is n, and the number of differentially expressed genes belonging to M is k. P-value of the hypergeometric distribution test is calculated to determine whether k/n in each subcategory of KEGG is significantly higher than M/N. q-value is an estimation for false discovery rate control. p.adjust is Benjamini-Hochberg adjusted p-value for multiple hypothesis testing. For heat maps in Fig. 2i-k and m, a two-tailed *t* test is used to assess whether a gene is differentially expressed.

### Cell lines and culture

HEK293T cells were cultured in Dulbecco’s Modified Eagle’s Medium (DMEM) containing 4.5 g/L glucose, supplemented with 10% fetal bovine serum (FBS) and 1% penicillin/streptomycin. Cultures were maintained in a humidified incubator at 37 °C with 5% CO2.

### Transfection, cell lysis, IP and immunoblotting

Approximately 1×10^6^ cells were seeded in each 6 cm dish and immediately transfected with 1 ng of p3.3-wrt-2/5/6-Flag combined with 1 ng of p3.3-ptr-8/16-HA plasmids using a PEIMax (Polysciences, 24765) transfection reagent. After 48 h, cells were lysed by adding 1 mL of Triton X-100 Lysis Buffer supplemented with protease and phosphatase inhibitors to each dish. The cell lysates were then collected and cleared by centrifugation at 20,000 *g* for 10 min at 4 °C.

For samples intended for immunoblottings, supernatants were mixed with 4× Laemmli sample buffer (Bio-Rad) and boiled for 5 min at 95 °C. For samples destined for immunoprecipitations (IP), supernatants were incubated with pre-washed Flag, HA, or agarose beads for 3 h at 4 °C with gentle rotation. The beads were subsequently washed three times with IP lysis buffer containing 500 mM NaCl, resuspended in 2× Laemmli sample buffer, and boiled at 95 °C for 5 min. Samples were then resolved by SDS-PAGE and analyzed by immunoblotting.

### siRNA knockdown

For siRNA knockdown experiments, we utilized RNAiMAX (Thermo Scientific, 13778-150) transfection reagent to transfect siRNAs into cells. 8 h post-transfection, the culture medium was replaced with fresh DMEM. In experiments involving transfection of both siRNAs and cDNAs, cDNAs were first transfected into the cells, followed by the transfection of siRNAs. Cells were harvested 48 h after siRNA transfection. The sgRNA oligos used were:

si*ptr-8*-sense-1: CCCUCCAACCAUUCCUCAUTT

si*ptr-8*-antisense-1: AUGAGGAAUGGUUGGAGGGTT

si*ptr-8*-sense-2: GCGGCUAUAUUCAUGGAUATT

si*ptr-8*-antisense-2: UAUCCAUGAAUAUAGCCGCTT

si*ptr-8*-sense-3: GCUCAUGUCGCAUUCCAUUTT

si*ptr-8*-antisense-3: AAUGGAAUGCGACAUGAGCTT

si*ptr-16*-sense-1: GGAAGACCCGUUUGUGCUUTT

si*ptr-16*-antisense-1: AAGCACAAACGGGUCUUCCTT

si*ptr-16*-sense-2: GCACCACAUUAAUGUCUAUTT

si*ptr-16*-antisense-2: AUAGACAUUAAUGUGGUGCTT

si*ptr-16*-sense-3: GCAAGAACUCAACCUCAAUTT

si*ptr-16*-antisense-3: AUUGAGGUUGAGUUCUUGCTT

### Mouse husbandry

Experiments were conducted with prior approval from the Institutional Animal Care and Use Committee (IACUC) at Peking University, IACUC number is FT-LiuY-8. Seven-month-old wild-type (WT) female and male C57BL/6 J mice were obtained from Biocytogen Pharmaceuticals (Beijing, China). All mice were maintained on a 12-h light/dark cycle at 25 ± 2 °C and relative humidity (50%). Food and water were provided *ad libitum*.

### Oligomycin resistance assay

Seven-month-old C57BL/6 J female and male mice were randomly assigned to experimental groups. Starting from day 1, mice received daily intraperitoneal injections of Itraconazole (MedChemExpress, R51211) at a concentration of 10 mg/kg; control mice were administered a vehicle solution consisting of 0.1% DMSO in Sulfobutylether-β-Cyclodextrin (MedChemExpress, HY17031). Beginning on day 5, all mice were injected intraperitoneally with oligomycin (MedChemExpress, HY-N6782) at a concentration of 0.5 mg/kg once a day. Throughout the experiment, the weight of each mouse was recorded every 7 days. Any animals that died were censored.

### Serum AMH and testosterone measurement

For AMH measurements, blood samples were drawn via the retro-orbital sinus using a serum separator tube (SST). Samples were allowed to clot for 2 h at room temperature before being centrifuged for 15 min at 1000 *g*. The serum was then stored at −80 °C until analysis. Serum AMH levels were measured using an enzyme-linked immunosorbent assay (ELISA) kit (CUSABIO, CSB-E13156m).

For testosterone measurements, 100 mg of testis tissue was rinsed with PBS, homogenized in 1 ml of PBS, and then centrifuged for 5 min at 5000 *g*, at 4 °C. The supernatants were aliquoted and stored at −80 °C. Tissue testosterone levels were measured using an ELISA kit (CUSABIO, CSB-E05101m).

### Fertility test

Seven-month-old C57BL/6J female and male mice were randomly assigned to experimental groups. Starting on day 1, mice were injected intraperitoneally once a day with Itraconazole (MedChemExpress, R51211) at a concentration of 10 mg/kg; control mice received a vehicle of 0.1% DMSO in Sulfobutylether-β-Cyclodextrin (MedChemExpress, HY17031). One week after beginning the Itraconazole injections, mating trials were initiated. During these trials, one Itraconazole-injected female mouse was placed in a cage with one Itraconazole-injected male for one week. Similarly, one control female mouse was paired with one control male for one week. Approximately three weeks later, the number of offspring per female was counted.

### Liver RNA isolation and cDNA synthesis

Seven-month-old C57BL/6J female and male mice were treated as indicated. Total RNA was isolated from the livers of these mice using TransZol Up reagent (TransGen Biotech, #ET111-01). The isolated RNA was then converted into cDNA using the EasyScript® One-Step gDNA Removal and cDNA Synthesis SuperMix (TransGen Biotech, #AE311-02) kits. This cDNA was used to detect the expression of hedgehog pathway-related genes.

### Correlation analyses

For genetic correlation analyses, GeneNetwork (http://www.genenetwork.org) was used to download and analyze datasets. Pearson’s r was employed to measure the correlations.

### Statistics and reproducibility

All statistical analyses were performed with GraphPad Prism 9.0. All survival curves were estimated with the log-rank (Mantel–Cox) test. For bar graphs and dot graphs, the two-tailed *t* test was used to determine statistical significance. Results are presented as mean ± SD. For statistical analysis, quantitative data were obtained from at least three biologically independent experiments. For detailed information of each assay, please see figures or figure legends. All images shown in the figures are representative of at least three biologically independent experiments. All western blots were repeated at least twice with different samples.

### Reporting summary

Further information on research design is available in the Nature Portfolio Reporting Summary linked to this article.

## Supplementary information

Supplementary Information

Description of Additional Supplementary Files

Supplementary Data 1

Supplementary Data 2

Supplementary Data 3

Supplementary Data 4

Supplementary Data 5

Reporting Summary

## Source data

Source Data

Peer Review file

## Data Availability

The RNA-seq data generated in this study have been deposited in the GEO under accession code GSE265886. All data supporting the findings of this study are available within this paper and its Supplementary Information files. Source data are provided with this paper.
