# Supplementary material for: A germline-to-soma signal triggers an age-related decline of mitochondrial stress response
Source: Nat Commun. 2024 Oct 8;15:8723. doi: 10.1038/s41467-024-53064-0 (PMC11461804; doi:10.1038/s41467-024-53064-0)
Supplement: Supplementary file 1 — Supplementary Information [file 41467_2024_53064_MOESM1_ESM.pdf]

Supplementary Figures for

A germline-to-soma signal triggers an age-related decline of mitochondrial stress response

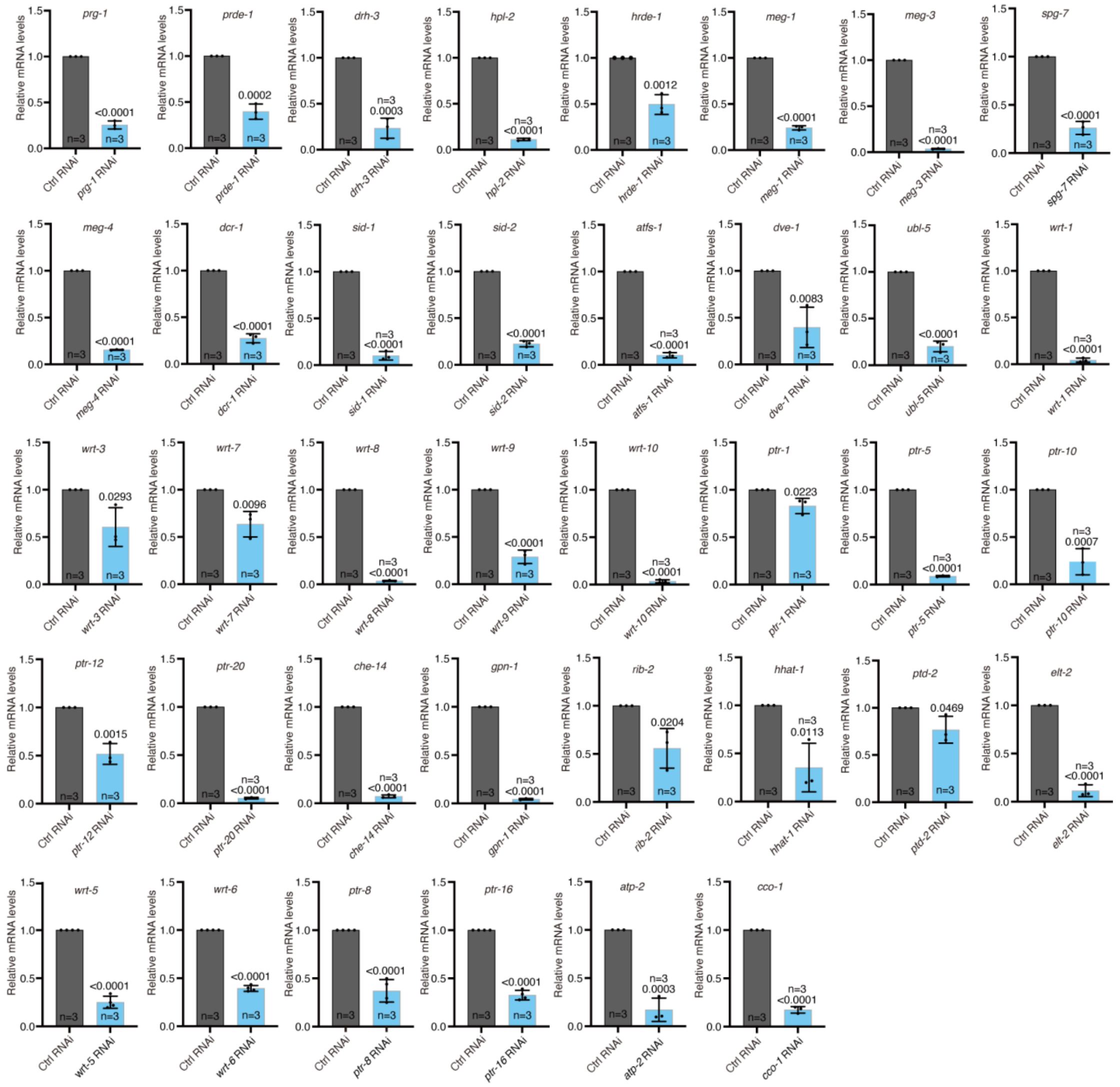

Supplementary Fig. 1 Verification of RNA interference (RNAi) efficiency. Relative mRNA levels were analyzed by qPCR. Error bars indicate mean ± SD. n refers to independent experiments. *p* values were assessed using a two-tailed *t*-test. RNAi treatment began at the L1 stage for all figure panels, unless otherwise specified. Source data are provided as a Source Data file.

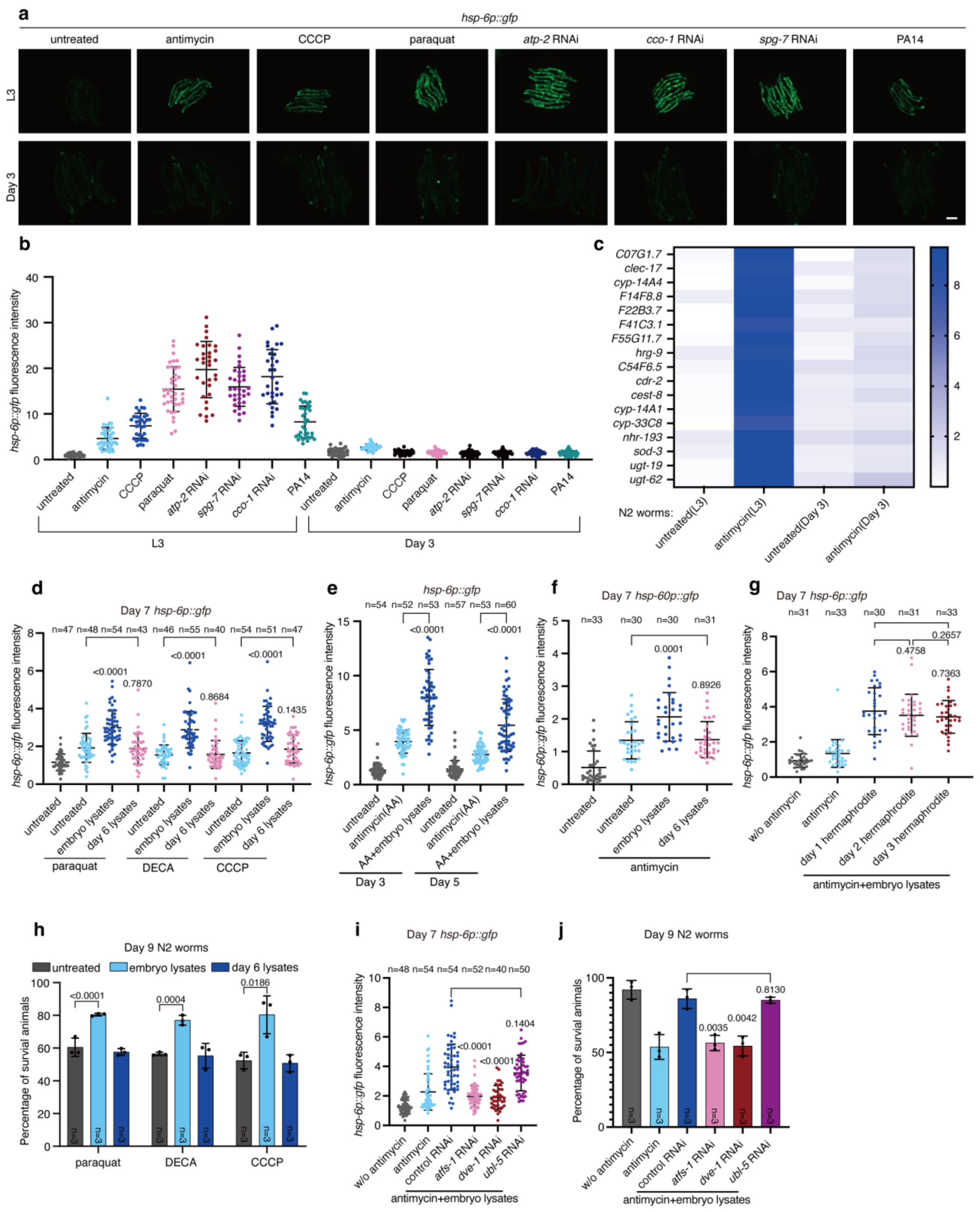

Supplementary Fig. 2 Age-related decline of UPR^{mt}. **a**, Representative images of *hsp-6p::gfp* worms treated with antimycin, paraquat, CCCP, PA14 (24h), or *atp-2*, *cco-1*, *spg-7* RNAi (48h) from the indicated stages. Scale bar, 200 µm. **b**, Quantification of GFP fluorescence intensity in panel a. **c**, qRT-PCR analysis of mitochondrial stress response genes in L3 and day 3 adult N2 worms treated with or without antimycin. Blue indicates a higher expression. The intensity of the color corresponds to the fold changes. **d**, Quantification of GFP fluorescence intensity in day 7 *hsp-6p::gfp* worms treated with the indicated drugs and/or lysates for 24h. **e, f**, Quantification of GFP fluorescence in *hsp-6p::gfp* worms treated with antimycin and

embryo lysates on day 3, 5 (e), or day 7 (f) of adulthood for 24h. **g**, Quantification of GFP fluorescence intensity in *hsp-6p::gfp* worms under the indicated treatments. Treatments with antimycin and embryo lysates were carried out on day 7 of adulthood for 24h. Embryos were harvested from day 1, 2, or 3 adult worms. **h**, Survival rate of adult day 9 N2 worms after 72h exposure to paraquat, DECA, or CCCP in the presence or absence of embryo/day 6 worm lysates. **i**, Quantification of GFP fluorescence in *hsp-6p::gfp* worms treated with RNAi starting on day 1 of adulthood, and antimycin and embryo lysates on day 7 for 24h. **j**, Survival rate of day 9 N2 worms exposed to antimycin and embryo lysates for 72h, with RNAi treatment starting on day 1 of adulthood. Error bars indicate mean ± SD. n represents the number of independent experiments for panels h and j, and the number of worms for panels d-g and i. *p* values were assessed using a two-tailed *t*-test. All images shown in the figures are representative of at least three biologically independent experiments. Source data are provided as a Source Data file.

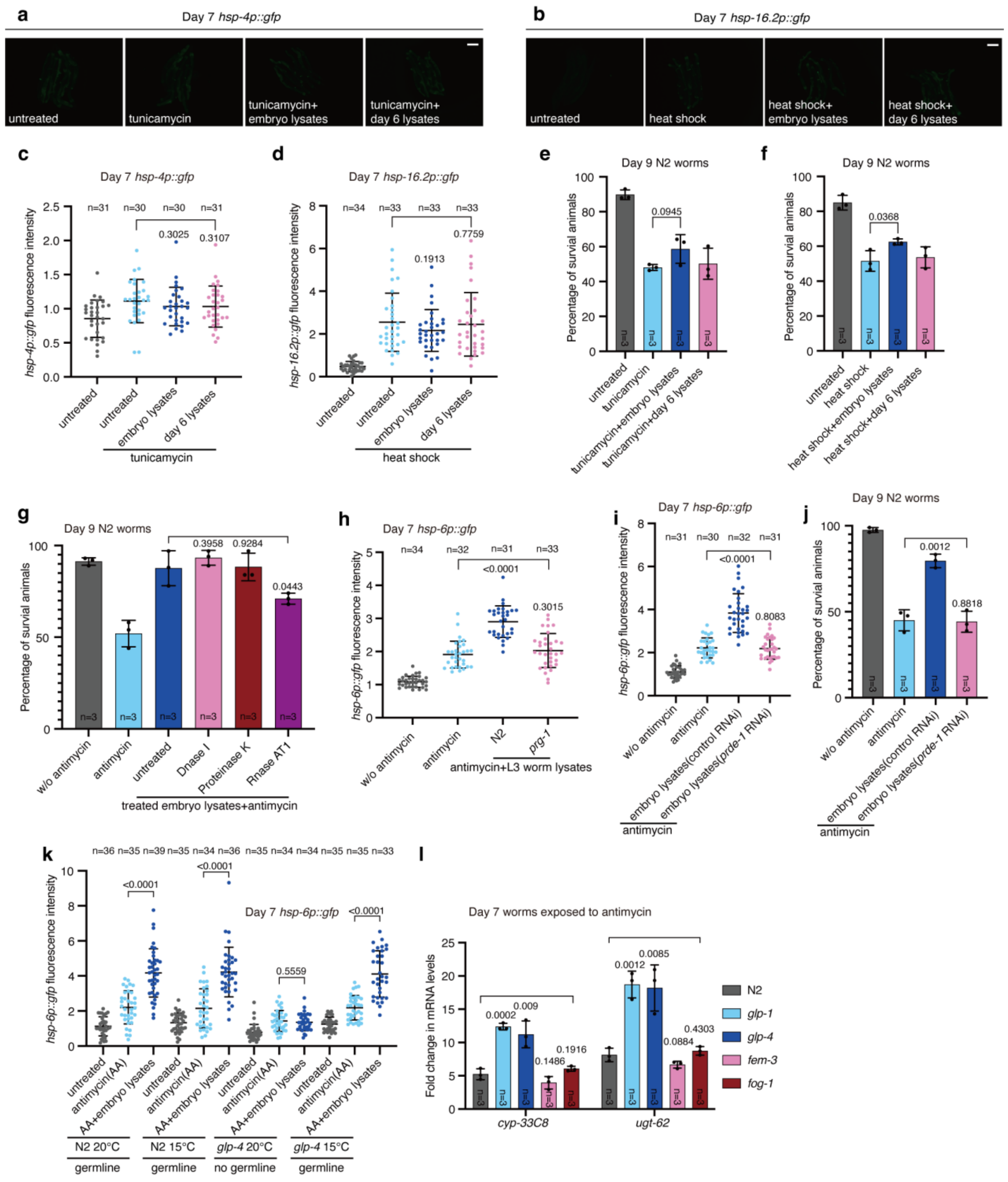

Supplementary Fig. 3 Treating adult worms with embryo lysates enables UPR^{mt} activation. a, b, Representative fluorescence images of day 7 *hsp-4p::gfp* (a) or *hsp-16.2p::gfp* (b) worms under indicated treatments. Scale bar, 200 µm. **c, d**, Quantification of GFP fluorescence intensity in panels a and b. **e, f**, Survival rate of day 9 N2 worms after 72h exposure to tunicamycin (e) or heat shock (f) with or without embryo/day 6 worm lysates. **g**, Survival rate of day 9 N2 worms after 72h antimycin exposure with embryo lysates treated with DNase I, Proteinase K, or RNase AT1. **h**, Quantification of GFP fluorescence intensity in *hsp-6p::gfp* worms treated with antimycin and L3 worm lysates on day 7 of adulthood for 24h. **i**, Quantification of GFP fluorescence in *hsp-6p::gfp* worms treated with antimycin and embryo lysates on day 7 for 24h. **j**, Survival rate of day 9 N2 worms after 72h exposure to antimycin and embryo lysates. **k**, GFP fluorescence quantification in *hsp-6p::gfp* or *glp-4; hsp-6p::gfp* worms

with or without antimycin and embryo lysates on day 7 for 24h. **l**, qRT-PCR analysis of UPR^{mt} mRNA levels in day 7 worms treated with antimycin for 24h. Error bars indicate mean ± SD. n represents the number of independent experiments for panels e, f, g, j and l, and the number of worms for panels c, d, h, i, and k. *p* values were assessed using a two-tailed *t*-test. All images shown in the figures are representative of at least three biologically independent experiments. Source data are provided as a Source Data file.

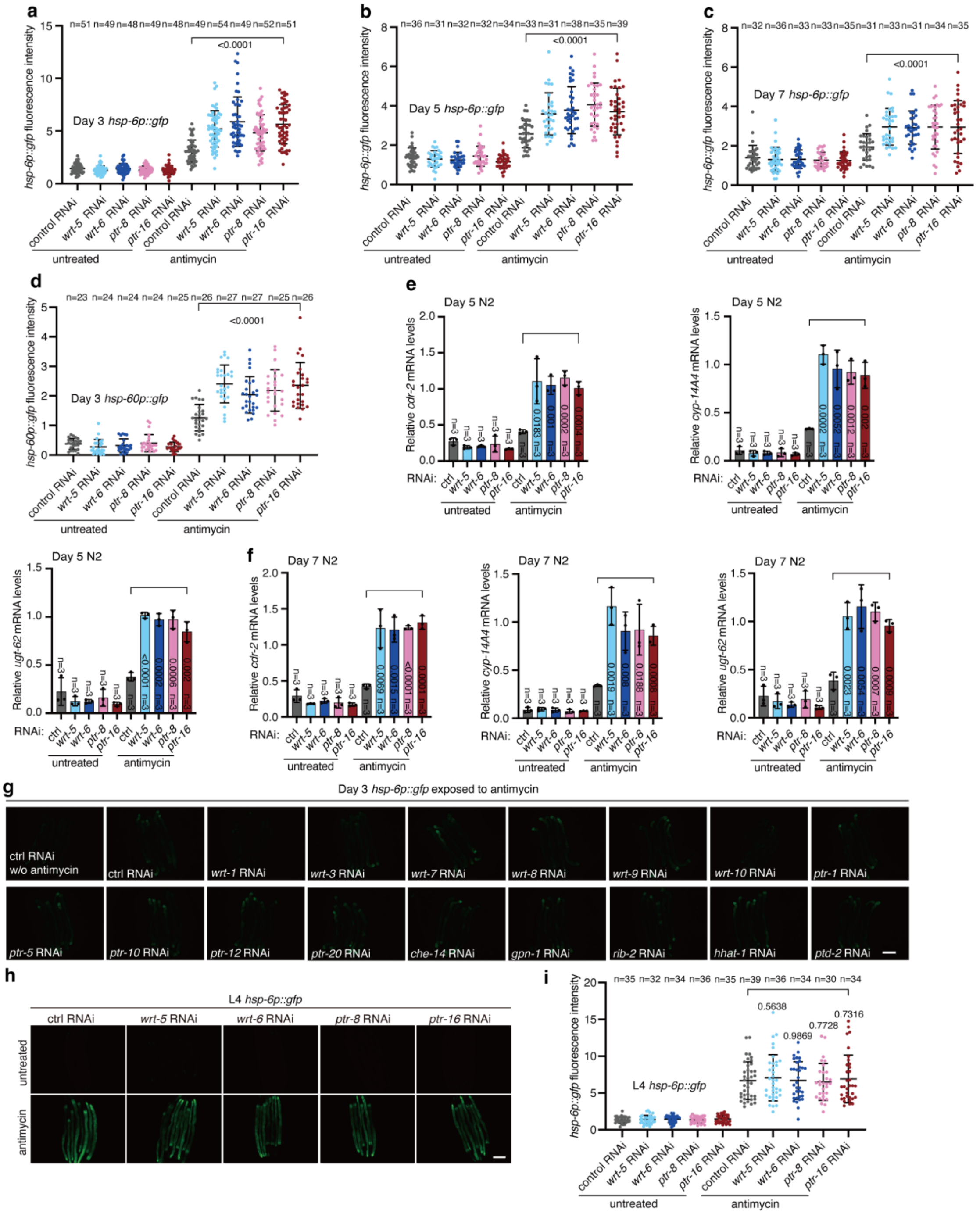

Supplementary Fig. 4 A hedgehog-related signal suppresses UPR^{mt} in adult worms. a-c, Quantification of GFP fluorescence intensity in *hsp-6p::gfp* worms on adult day 3 (a), 5 (b), and 7 (c) treated with antimycin for 24h. **d**, Quantification of GFP fluorescence intensity in day 3 *hsp-60p::gfp* worms treated with antimycin for 24h. **e, f**, qRT-PCR analysis of the indicated UPR^{mt} genes in day 5 (e) and day 7 (f) wild-type (N2) adults exposed to antimycin. **g, h**, Representative fluorescence images of day 3 (g) or L4 (h) *hsp-6p::gfp* worms treated with antimycin for 24h. Scale bar, 200 µm. **i**, Quantification of GFP fluorescence intensity in panel h. Error bars indicate mean ± SD. n represents the number of independent experiments for panels e and f, and the number

of worms for panels a-d and i. *p* values were assessed using a two-tailed *t*-test. All images shown in the figures are representative of at least three biologically independent experiments. Source data are provided as a Source Data file.

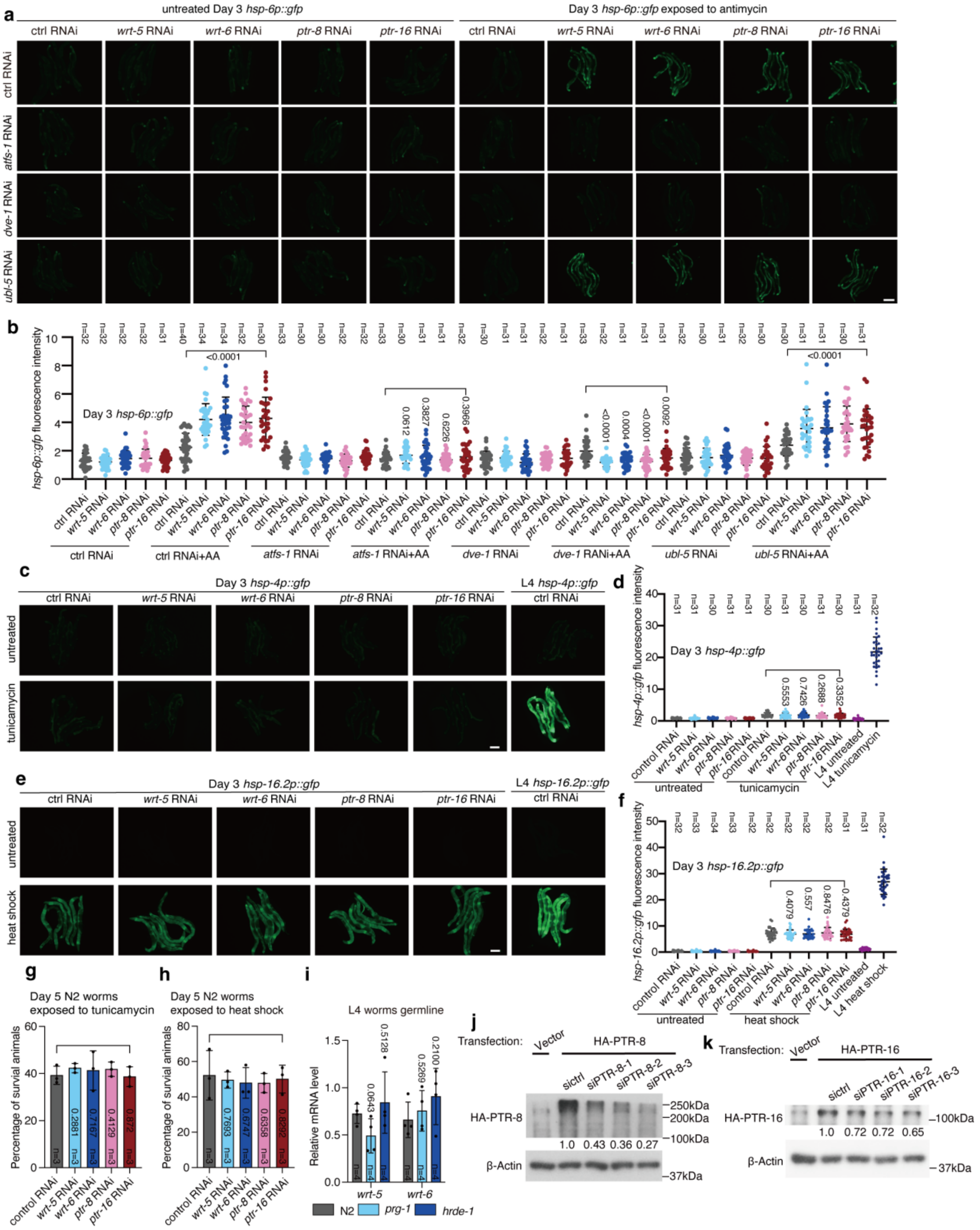

Supplementary Fig. 5 WRT-5/6-mediated hedgehog-related signal has no effect on UPR^{ER} and HSR in adult worms. a, Representative fluorescence images of adult day 3 *hsp-6p::gfp* worms treated with antimycin for 24h. Scale bar, 200 µm. **b**, Quantification of GFP fluorescence intensity in panel a. **c**, Representative fluorescence images of day 3 *hsp-4p::gfp* worms treated with tunicamycin for 24h. Scale bar, 200 µm. **d**, Quantification of GFP fluorescence intensity in panel c. **e**, Representative fluorescence images of day 3 *hsp-16.2p::gfp* worms treated with heat shock. Scale bar, 200 µm. **f**, Quantification of GFP fluorescence intensity in panel e. **g, h**, Survival rate of day 5 wild-type (N2) worms after 72h exposure to tunicamycin (g) or exposure to heat shock (h). **i**, qRT-PCR analysis of *wrt-5* and *wrt-6* expression in the germline of L4

stage worms. **j, k**, Immunoblotting of PTR-8 (j) and PTR-16 (k). Immunoblot of lysates from HEK 293T cells treated with siCtr or siPTR-8/16. Error bars indicate mean ± SD. n represents the number of independent experiments for panels g, h and i, and the number of worms for panels b, d, and f. *p* values were assessed using a two-tailed *t*-test. All images shown in the figures are representative of at least three biologically independent experiments. All western blots were repeated at least twice with different samples. Source data are provided as a Source Data file.

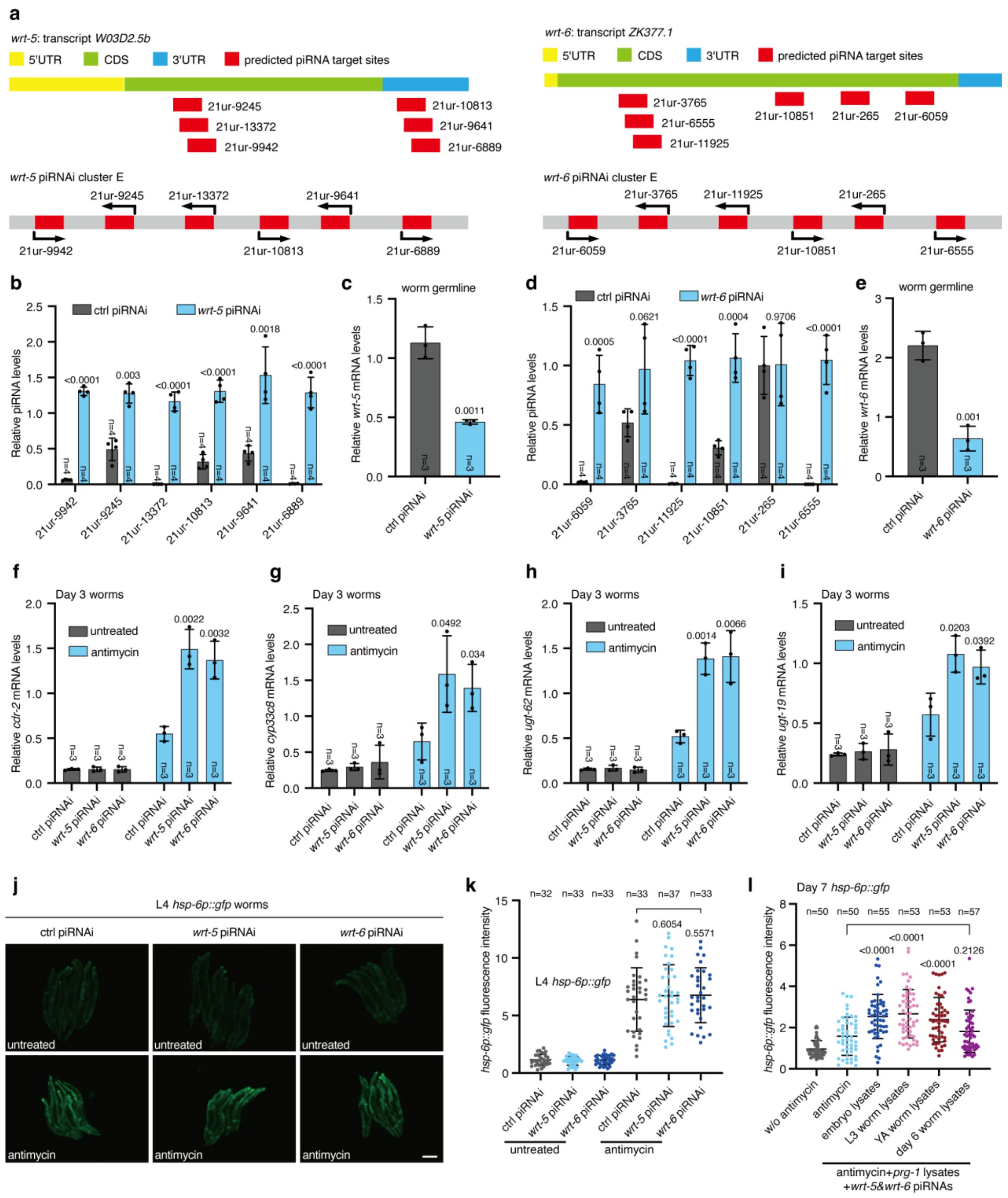

Supplementary Fig. 6 piRNAs targeting *wrt-5* and *wrt-6* block UPR^{mt} and mitochondrial stress resistance attenuation. a, Sequences and predicted binding sites of *wrt-5* (left) and *wrt-6* (right) targeting piRNAs, which were added to the synthetic piRNAi cluster E. **b, d**, qRT-PCR analysis of piRNAs targeting *wrt-5* (b) and *wrt-6* (d) in L4 worms. **c, e**, qRT-PCR analysis of *wrt-5* (c) and *wrt-6* (e) mRNA levels in the germlines of L4 worms. **f-i**, qRT-PCR analysis of UPR^{mt} genes mRNA levels in day 3 worms with overexpression of piRNAs targeting *wrt-5*, *wrt-6*, or a control gene. Antimycin treatment occurred on day 3 of adulthood for 24h. **j**, Representative fluorescence images of *hsp-6p::gfp* worms with overexpression of piRNAs targeting *wrt-5*, *wrt-6*, and a control gene. Antimycin treatment occurred on L4 for 24h. Scale bar, 200 μm. **k**, Quantification of GFP fluorescence intensity in panel j. **l**, Quantification of GFP fluorescence intensity in *hsp-6p::gfp* worms with the indicated treatments.

Antimycin, *prg-1* embryo or worm lysates, and piRNAs were provided to day 7 adult worms for 24h. Error bars indicate mean ± SD. n represents the number of independent experiments for panels b-i, and the number of worms for panels k and l. *p* values were assessed using a two-tailed *t*-test. All images shown in the figures are representative of at least three biologically independent experiments. Source data are provided as a Source Data file.

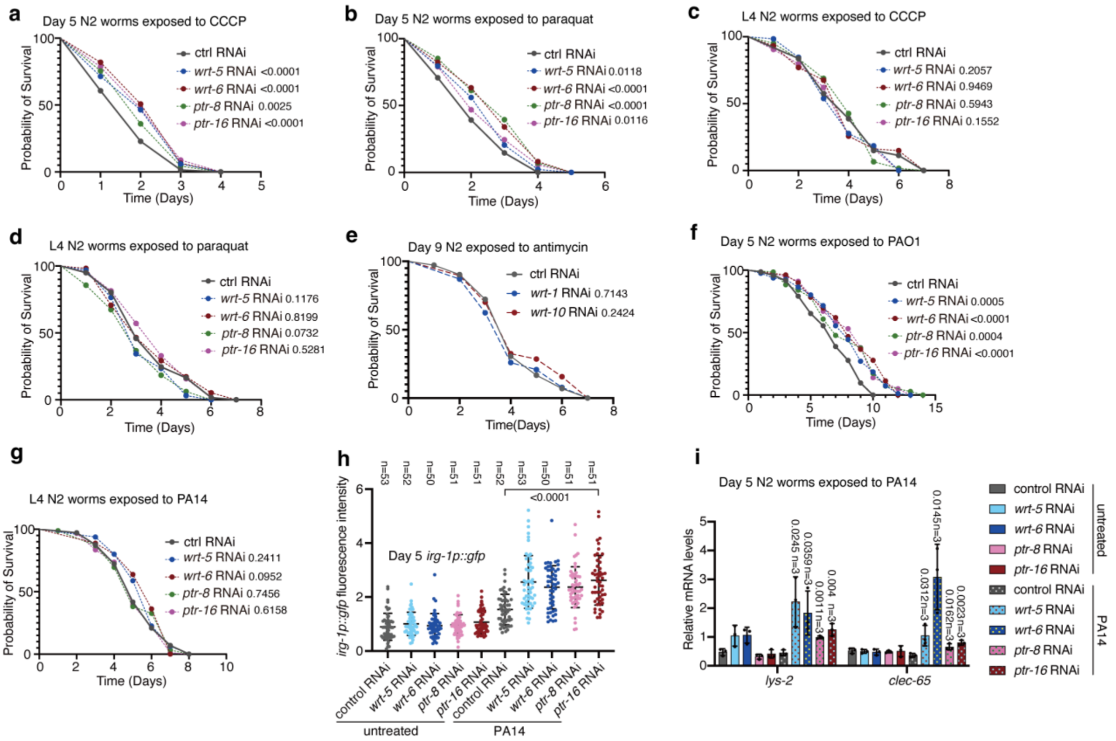

Supplementary Fig. 7 Suppression of the hedgehog-related signaling promotes the survival of adult worms under mitochondrial stress or pathogen infection. a-g, Survival curves of wild-type (N2) worms at indicated developmental stages, raised in the presence or absence of RNAi targeting hedgehog-related genes, under specified treatments. **h**, Quantification of GFP fluorescence intensity in *irg-1p::gfp* worms with the indicated treatments. PA14 infection occurred on day 5 of adulthood for 48h. **i**, qRT-PCR analysis of the indicated innate immune response genes in day 5 N2 animals exposed to PA14. Error bars indicate mean ± SD. n represents the number of independent experiments for panel i, and the number of worms for panel h. *p* values were assessed using the Log-rank (Mantel–Cox) test for panels a-g, and a two-tailed *t*-test for panels h and i. Source data are provided as a Source Data file.

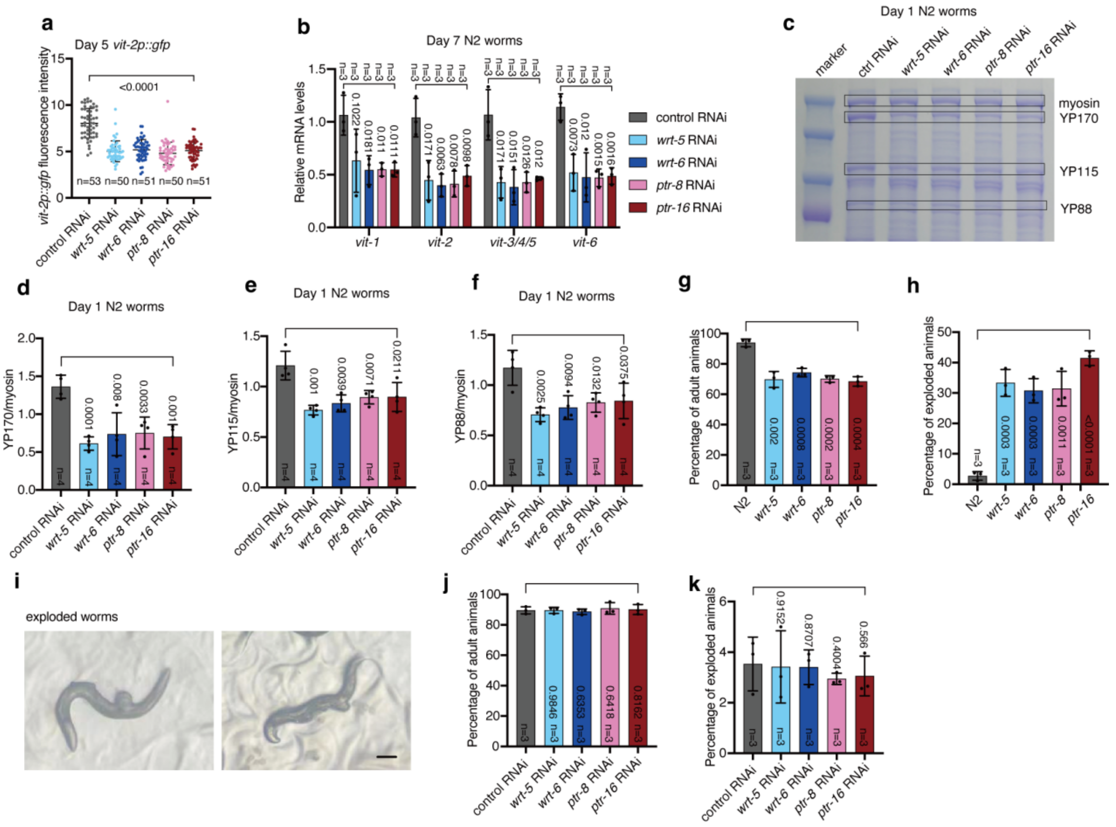

Supplementary Fig. 8 Suppression of the hedgehog-related signaling impairs development and fertility. a, Quantification of GFP fluorescence intensity in day 5 *vit-2p::gfp* adults with the indicated RNAi. **b**, qRT-PCR analysis of the *vit* family genes in day 7 N2 adults treated with the indicated RNAi. **c**, Representative Coomassie stained gels showing yolk protein (YP) levels in day 1 N2 worms. **d-f**, Quantification of Yolk Protein Proportion in day 1 N2 worms. **g**, Hedgehog pathway-related gene mutants display growth delay, as indicated by the percentage of adult worms. **h**, Hedgehog pathway-related gene mutants display the exploded (ruptured vulva) phenotype more frequently, based on the percentage of exploded worms at day 5 of adulthood. **i**, Representative images of exploded worms. Scale bar, 200 µm. **j, k**, Hedgehog pathway-related genes RNAi in N2 worms does not display growth delay (j) or higher frequency of exploded phenotype (k). Error bars indicate mean ± SD. n represents the number of independent experiments for panels b, d-h, j, and k, and the number of worms for panel a. *p* values were assessed using a two-tailed *t*-test. All images shown in the figures are representative of at least three biologically independent experiments. Source data are provided as a Source Data file.

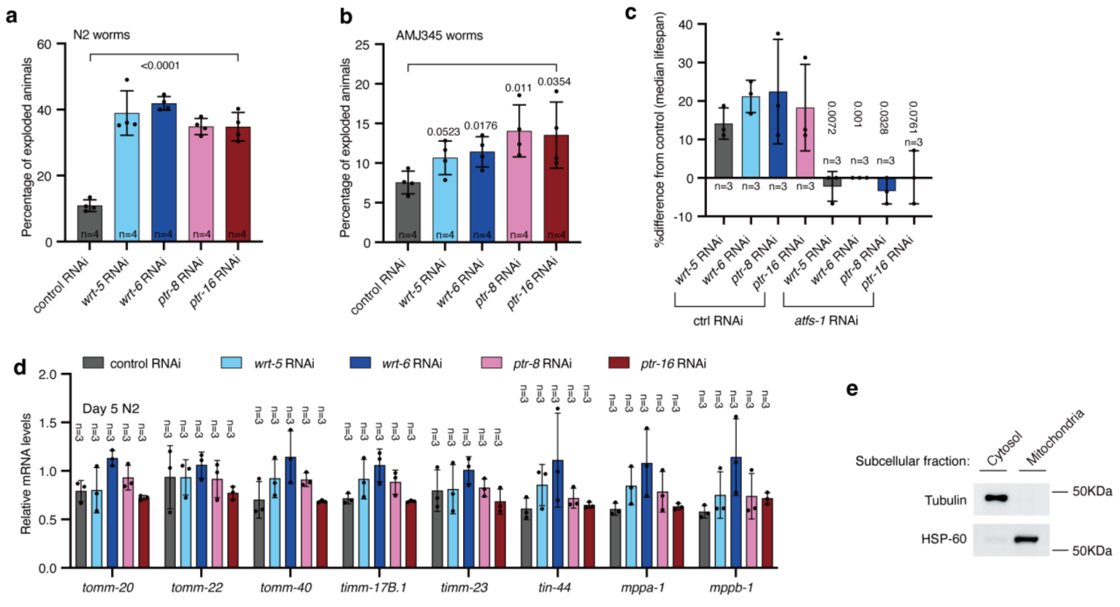

Supplementary Fig. 9: Suppressing a hedgehog-like signal extends lifespan. a, b, Hedgehog pathway-related gene RNAi in wild-type (N2) worms (a) or AMJ345 worms (b) results in a higher frequency of the exploded (ruptured vulva) phenotype, based on the percentage of exploded worms throughout their lifespan. **c**, Median lifespan extension of AMJ345 worms under the indicated treatments. **d**, qRT-PCR analysis of the indicated mitochondrial import machinery genes in day 5 N2 worms. **e**, Immunoblotting of HSP-60 and tubulin in lysates collected from subcellular fractionation. Error bars indicate mean ± SD. n represents the number of independent experiments for panels a-c. *p* values were assessed using a two-tailed *t*-test. All western blots were repeated at least twice with different samples. Source data are provided as a Source Data file.

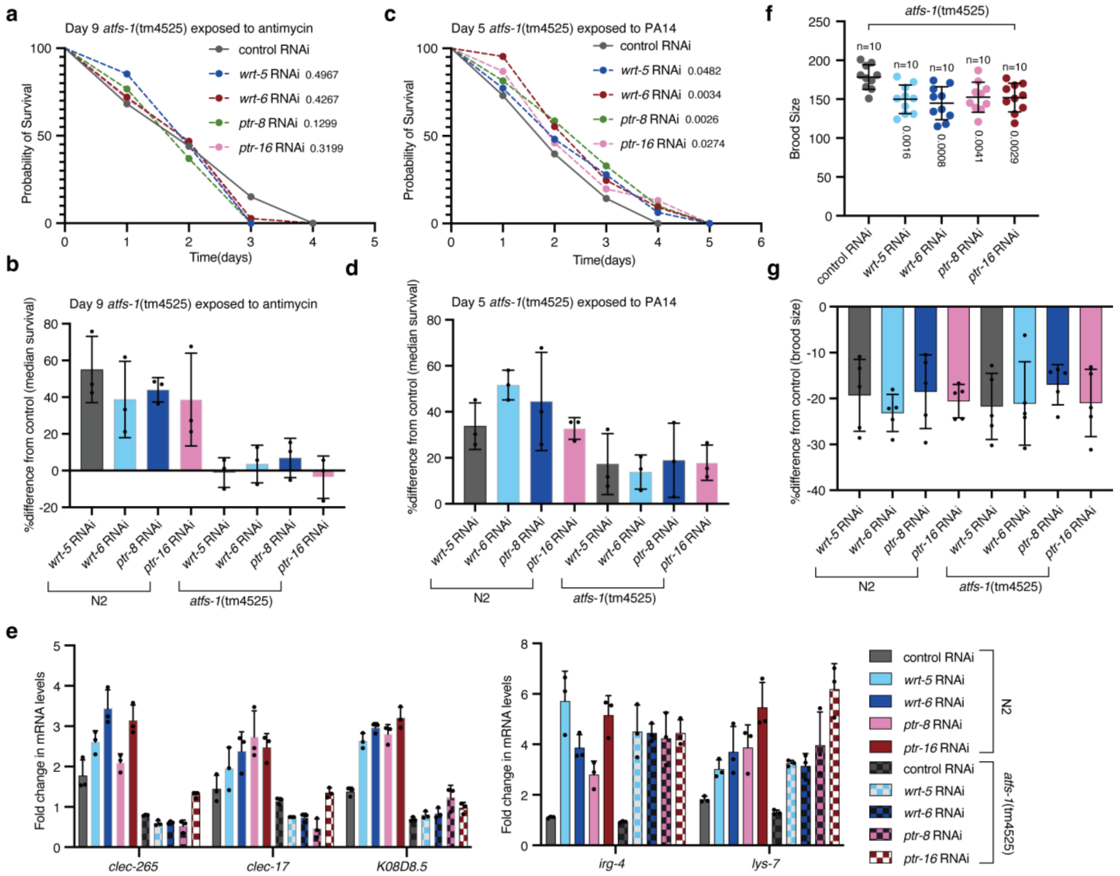

Supplementary Fig. 10 Suppression of the hedgehog-like signal in *atfs-1* mutants increases resistance to pathogen infection but not to mitochondrial stress, and reduces brood size. a, c, Survival curves of day 9 *atfs-1* mutants exposed to antimycin (a) or PA14 (c). **b, d**, Median survival extension of day 9 *atfs-1* mutants exposed to antimycin (b) or PA14 (d). **e**, qRT-PCR analysis of indicated innate immune response genes in day 5 wild-type (N2) worms and *atfs-1* mutants exposed to PA14 for 48h. **f**, Brood size of *atfs-1* mutants treated with the indicated RNAi from the L1 stage. **g**, Brood size reduction in *atfs-1* mutant worms. Error bars indicate mean ± SD. n represents the number of independent experiments for panel f. *p* values were assessed using the Log-rank (Mantel–Cox) test for panels a and c, and a two-tailed *t*-test for panel f. Source data are provided as a Source Data file.

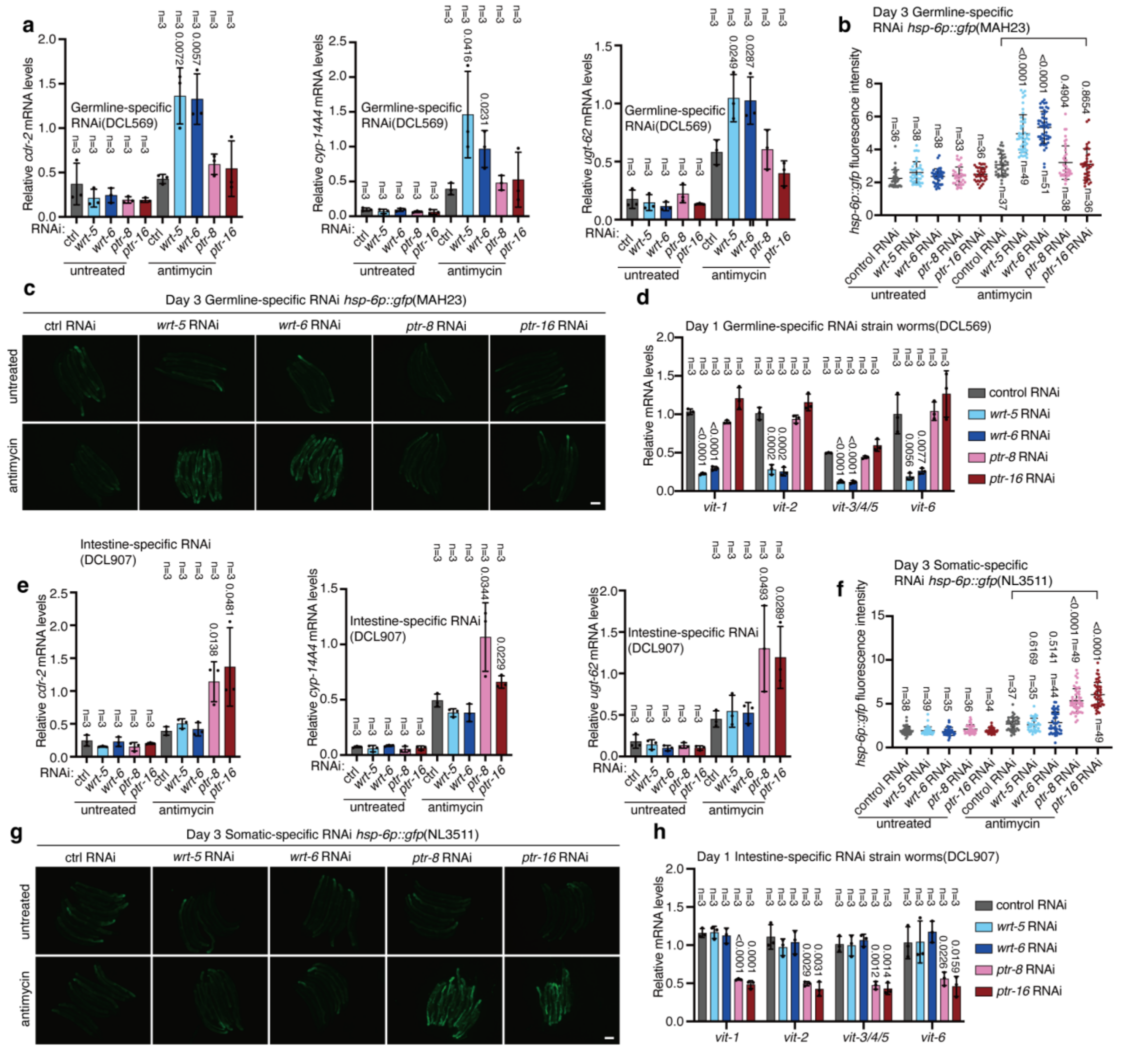

Supplementary Fig. 11 A germline-to-soma hedgehog-like signal allows adult worms to invest their resources in reproduction rather than somatic maintenance. **a, e**, qRT-PCR analysis of mRNA levels of indicated UPR^{mt} genes in day 3 germline- (a) and intestine-specific (e) RNAi strain treated with RNAi and antimycin. Antimycin treatment occurred on day 3 of adulthood for 24h. **b, f**, Quantification of GFP fluorescence intensity in day 3 germline- (b) and somatic-specific (f) RNAi *hsp-6p::gfp* reporter strains treated with antimycin for 24h. **c, g**, Representative fluorescence images of the indicated worms. Antimycin treatment occurred on day 3 of adulthood for 24h. Scale bar, 200 µm. **d, h**, qRT-PCR analysis of the *vit* family genes in day 1 germline- (d) and intestine-specific (h) RNAi strain under the indicated treatments. Error bars indicate mean ± SD. n represents the number of independent experiments for panels a, d, e, and h, and the number of worms for panels b and f. *p* values were assessed using a two-tailed *t*-test. All images shown in the figures are representative of at least three biologically independent experiments. Source data are provided as a Source Data file.

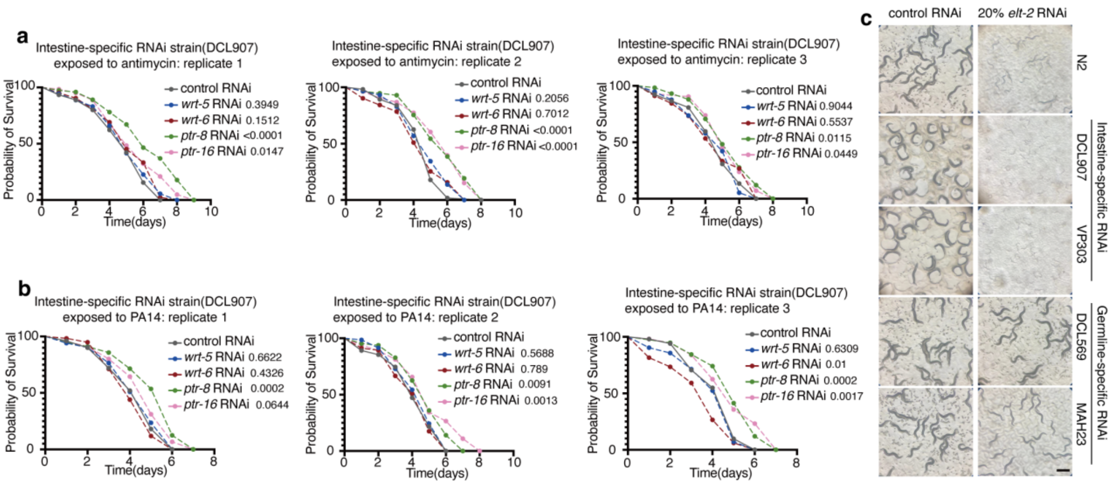

Supplementary Fig. 12 A germline-to-soma hedgehog-like signal allows adult worms to invest their resources in reproduction rather than somatic maintenance **a, b**, Survival rate of day 9 intestine-specific RNAi strain treated with the indicated RNAi and exposed to antimycin (a) or PA14 (b). Data from 3 biological replicates are shown. **c**, Representative images of N2, intestine- or germline-specific RNAi strain under the indicated treatments. Scale bar, 500 µm. *p* values were assessed using the Log-rank (Mantel–Cox) test. All images shown in the figures are representative of at least three biologically independent experiments. Source data are provided as a Source Data file.

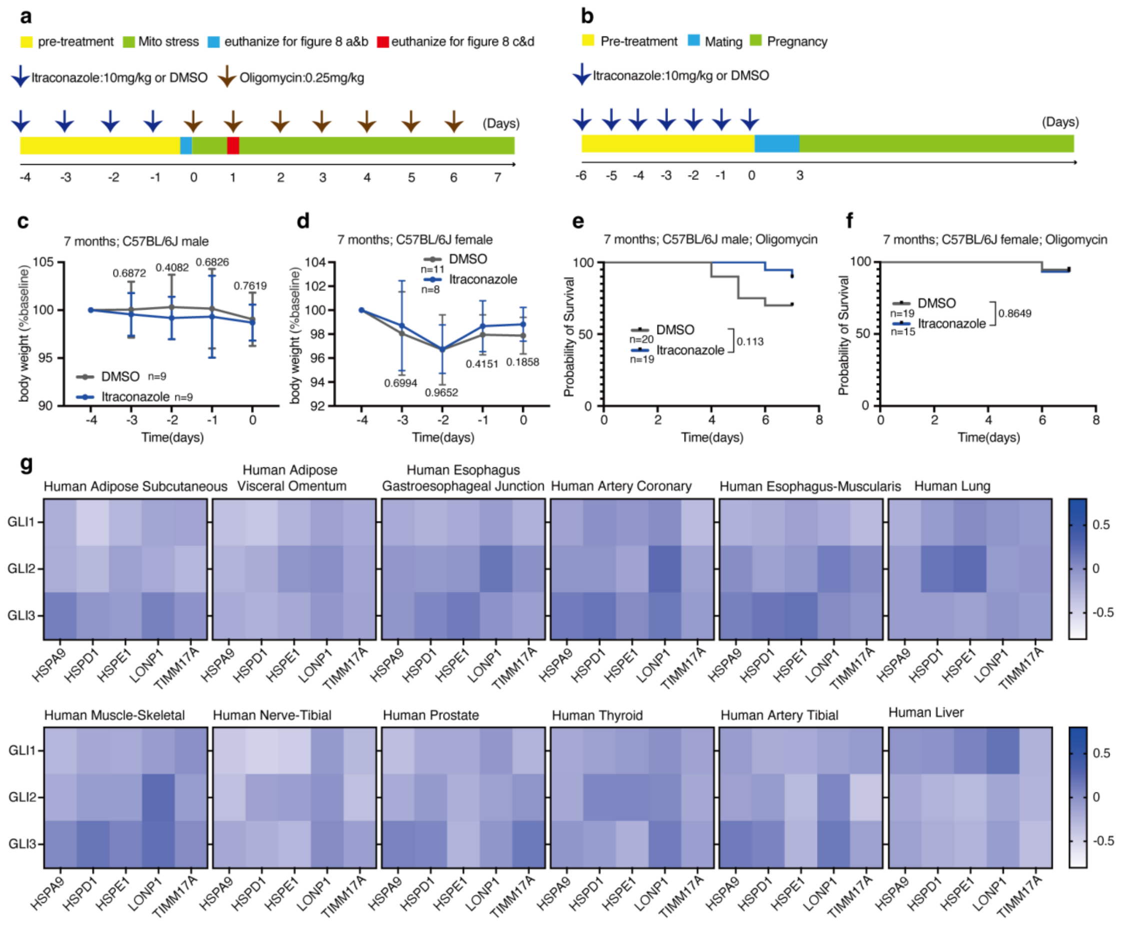

Supplementary Fig. 13 Suppression of the hedgehog-like signal increases resistance to mitochondrial stress but reduces fertility in mice. a, b, Workflow diagram for the oligomycin treatment assay (a) and fertility assay (b) conducted in mice. **c, d**, Weight loss in seven-month-old C57BL/6J male (c) and female (d) mice during itraconazole treatment. **e, f**, Survival curves of seven-month-old C57BL/6J male (e) and female (f) mice during oligomycin treatment. **g**, Pearson's correlation of GLI1, GLI2, GLI3, and UPR^{mt} mRNA levels in various human tissues. Blue indicates a positive correlation and white indicates a negative correlation. The intensity of the colors corresponds to the correlation coefficient. Error bars indicate mean ± SD. n represents the number of mice for panels c-f. *p* values were assessed using the Log-rank (Mantel–Cox) test for panels e and f, and a two-tailed *t*-test for panels c and d. Source data are provided as a Source Data file.
