# Supplementary material for: A germline-to-soma signal triggers an age-related decline of mitochondrial stress response
Source: Nat Commun. 2024 Oct 8;15:8723. doi: 10.1038/s41467-024-53064-0 (PMC11461804; doi:10.1038/s41467-024-53064-0)
Supplement: Supplementary file 2 — Description of Additional Supplementary Files [file 41467_2024_53064_MOESM2_ESM.pdf]

Description of Additional Supplementary Files

File name: Supplementary Data 1
Description: GO annotations of differentially expressed genes in *C. elegans*.
The hypergeometric distribution test is applied to assess whether a known biological function or process is enriched for differentially expressed genes. In this context, all expressed genes are considered as the total background number (N), the total number of genes annotated to a particular subset of known gene sets (KEGG) is represented by M, the number of differentially expressed genes is denoted by n, and the number of differentially expressed genes associated with M is k. The *p*-value from the hypergeometric distribution test determines whether k/n in each KEGG subcategory is significantly higher than M/N. The q-value is used to estimate the false discovery rate, while the *p*.adjust value, based on the Benjamini-Hochberg method, adjusts for multiple hypothesis testing. Detailed methods are provided in the methods section.

File name: Supplementary Data 2
Description: Results of the candidate-based RNAi screening.
RNAi screening was conducted by feeding worms *Escherichia coli* HT115, with RNAi clones sourced from the Ahringer library. Antimycin was used to induce UPR^{mt}, and GFP expression was scored from ns (no induction) to +++ (strong induction). Detailed methods are provided in the Methods section.

File name: Supplementary Data 3
Description: List of *c.elegans* strains used in the work.

File name: Supplementary Data 4
Description: Sequences of quantitative real-time PCR primers.

File name: Supplementary Data 5
Description: List of RNAi clones in this study.
