# Supplementary material for: A germline-to-soma signal triggers an age-related decline of mitochondrial stress response
Source: Nat Commun. 2024 Oct 8;15:8723. doi: 10.1038/s41467-024-53064-0 (PMC11461804; doi:10.1038/s41467-024-53064-0)
Supplement: Supplementary file 5 — Supplementary Data 3 [file 41467_2024_53064_MOESM5_ESM.docx]

*C. elegans* strains used in this study.

| **Strains** | **Source** |
| --- | --- |
| N2 | CGC |
| SJ4100 (zcIs13[*hsp-6p::gfp*]) | CGC |
| CL2070 (dvIs70[*hsp-16.2p::gfp*]) | CGC |
| SJ4005 (zcIs4[*hsp-4p::gfp*]) | CGC |
| AU133(agIs17[*irg-1p::gfp*]) | CGC |
| SS104(*glp-4(bn2)*) | CGC |
| NL3511(*ppw-1(pk1425)*) | CGC |
| MAH23(*rrf-1(pk1417)*) | CGC |
| RB843(*wrt-5(ok670)*) | CGC |
| HRN680(*wrt-6(aus41)*) | CGC |
| VC3790(*F47D12.6(gk3750) III ;ptr-16(gk3752)V ;M163.11(gk3751)*) | CGC |
| AGD1032(*glp-1*(e2141)) | CGC |
| JK816 (*fem-3*(q20)) | CGC |
| JK560 (*fog-1*(q253)) | CGC |
| SX922 (*prg-1*(n4357)) | CGC |
| YY11(*dcr-1*(mg375)) | CGC |
| tm8237(*ptr-8*) | NBRP |
| tm4525(*atfs-1*) | NBRP |
| tm1200(*hrde-1*) | NBRP |
| BCN9071(*[vit-2](https://cgc.umn.edu/gene/243595)(crg9070[[vit-2](https://cgc.umn.edu/gene/243595)::gfp]*) ) | Dr. Meng-Qiu Dong |
| DCL906(*rde-1(mkc36) V; neIs9[myo-3::HA::rde-1 + pRF4(rol-6)]*) | Dr. Di Chen |
| DCL907(*rde-1(mkc36) V; kbIs7[Pnhx-2::rde-1 + pRF4(rol-6)]*) | Dr. Di Chen |
| DCL1023(*rde-1(mkc36); mkcSi98[col-12p::rde-1::col-12 3'UTR + unc-119(+)]*) | Dr. Di Chen |
| DCL569(*mkcSi13[sun-1p::rde-1::sun-1 3′UTR + unc-119(+)] II; rde-1(mkc36) V; zcIs18 [ges-1::GFP(cyt)]*) | Dr. Di Chen |
| NR350(*[rde-1](https://cgc.umn.edu/gene/241731)([ne219](https://cgc.umn.edu/variation/1887296)) V; [kzIs20](https://cgc.umn.edu/transgene/21862) [hlh-1p::rde-1 + sur-5p::NLS::GFP]*) | Dr. Qinghua Zhou |
| TU3401(*[sid-1](https://cgc.umn.edu/gene/243927)([pk3321](https://cgc.umn.edu/variation/1891778)) V; [uIs69](https://cgc.umn.edu/transgene/20373) [pCFJ90 (myo-2p::mCherry) + unc-119p::sid-1]*) | Dr. Qinghua Zhou |
| VP303(*[rde-1](https://cgc.umn.edu/gene/241731)([ne219](https://cgc.umn.edu/variation/1887296)) V; [kbIs7](https://cgc.umn.edu/transgene/23579) [nhx-2p::rde-1 + rol-6(su1006)]*) | Dr. Qinghua Zhou |
| AMJ345(*[rde-1](https://cgc.umn.edu/gene/241731)([ne219](https://cgc.umn.edu/variation/1887296)) V;jamSi2 [mex-5p::rde-1(+)]*) | Dr. Qinghua Zhou |
| PHX-YL-1(piRNA chromosomal array targeting *wrt-5*);SJ4100 (*zcIs13[hsp-6p::gfp]*) | SunyBiotech |
| PHX-YL-2(piRNA chromosomal array targeting *wrt-6*);SJ4100 (*zcIs13[hsp-6p::gfp]*) | SunyBiotech |
| PHX-YL-3(piRNA chromosomal array targeting control gene); SJ4100 (*zcIs13[hsp-6p::gfp]*) | SunyBiotech |
| PHX-YL-4(*Ppie-1-wrt-5 cDNA-tbb-2 3'UTR&Pmyo-2-mCherry*;SJ4100 (*zcIs13[hsp-6p::gfp]*)) | SunyBiotech |
| PHX-YL-5(*Ppie-1-wrt-6 cDNA-tbb-2 3'UTR&Pmyo-2-mCherry*;SJ4100 (*zcIs13[hsp-6p::gfp]*)) | SunyBiotech |
| PHX-YL-6(*Pges-1-ptr-8 cDNA-let-858 3'UTR&Pmyo-3-mCherry*; SJ4100 (*zcIs13[hsp-6p::gfp]*)) | SunyBiotech |
| PHX-YL-7(*Pges-1-ptr-8 cDNA-let-858 3'UTR&Pmyo-3-mCherry*; SJ4100 (*zcIs13[hsp-6p::gfp]*)) | SunyBiotech |
