# Supplementary material for: A germline-to-soma signal triggers an age-related decline of mitochondrial stress response
Source: Nat Commun. 2024 Oct 8;15:8723. doi: 10.1038/s41467-024-53064-0 (PMC11461804; doi:10.1038/s41467-024-53064-0)
Supplement: Supplementary file 6 — Supplementary Data 4 [file 41467_2024_53064_MOESM6_ESM.docx]

qPCR primer sequences in this study

| **Name** | **Primers (forward)** | **Primers (reverse)** |
| --- | --- | --- |
| *rpl-32* | ATGTTAAGGATTTGGACATGCTCCTC | TTCAGTGCGAAGACGAGCGTT |
| *wrt-5* | ATGTGCTCCATGTGGCTCAT | GTTCCGGGCATTCTGCATACTTC |
| *wrt-6* | TTGGATGTGAGTTGCGACCCT | CTGGTCCAAGGAAGATGCTCTTG |
| *ptr-8* | CGTCAAGAGTGACGAGGATATGG | GCGCTCACTTTTGGTGTTAATGC |
| *ptr-16* | CGGCCAGTGTAATGATAATCAGGTCA | CCGTTCGGAAGTAGAGAACGAC |
| *vit-1* | GAGGTTCGCTTTGACGGATA | GGCTTCACATTCCTCGTTCT |
| *vit-2* | GACACCGAGCTCATCCGCCCA | TTCCTTCTCTCCATTGACCT |
| *vit-3/4/5* | CATGTGCACCATCGAAGAACTC | CCAATGTGGTTTCAATGACAAGTTG |
| *vit-6* | TTCACCCAGAAGCCAGTTC | AGGATGGGAGGCAGTAGAC |
| *hsp-6* | CGCTGGAGATAAGATCATCGCTG | ATGGACGAGAGCGTGATCGAA |
| *cdr-2* | GTGATGACAAGCTTCGTTGGTCAAG | AGCAGCTGGTAGAGATGGGATAT |
| *cyp-14A4* | AATCTTCACCAGATTCCGGGTG | ATTGTTGAATTCGCCTTGCGTTAC |
| *ugt-62* | TGCACAGAAGAATGACCTCAAACG | CCTTTGCCAATGTCGGACGA |
| *irg-1* | CGGAGAAAAAGGAGAAGCTGCT | TTGCTCCTTTGTCATGCTCAGC |
| *clec-17* | CAACTCATGCAGACGACTGTACC | CCACACTGCTCCAATGAGGTAAT |
| *C07G1.7* | AAAGCTGAAGAAGCTTCAACCG | AGGAACACCTCGTTCGTAATCG |
| *F22B3.7* | AAATGCAACGGGAACTCTCTTTG | CCTGATCCATCTGCATGACCC |
| *F55G11.7* | AGGGAATCTTGTTCAAACTTCCCAAG | GCATAGCGATAACTTGACTCGTCAC |
| *F41C3.1* | ACTTGTTCAGAACTATGAGGACTTGC | AACGCTGGATTTGGATTCTCCC |
| *F14F8.8* | CAGTCCTTCTTGTTCTGGCTCTCT | AGCATTCATTGGCATAGTGTTTGCT |
| *hrg-9* | AACTGAAGGATGAGTACCATGTCG | AGCATCTGGATACCATTGTGTCGTA |
| *cyp-33C8* | TGGATGGGCAAAGACCCATATGT | ATCTACGATGGGTGTTCCAAACATG |
| *cyp-14A1* | TTCACCAGTTCCCTCCAGACAATG | CGGTAAGTGAATGTGTCACCTTGATT |
| *ugt-19* | GTGTGCATATCCATTCTTCGAAGCC | ATCTTTGCCACATAGTCATCCGG |
| *cest-8* | GTGAAAAGTGCTTGAAGTCCAAGTG | AACTCCCAATCTAACTGCAGGT |
| *C54F6.5* | ATGTTCACCAAGACCCTCCTCC | GAGCAAACACAGTCTCCGGTGA |
| *sod-3* | ATCTTGAAGATCGCCACCTGT | CCAGTTGGCAATCTTCCAAATAGCAT |
| *nhr-193* | GGAATGACAATTGAACATTTCCAGCAC | TTCCGATAAGATTCCTGGGGAACAC |
| *clec-265* | CACCACACCCCTCACGTATG | GAGAATCTGGGCATGGCTGA |
| *K08D8.5* | TGTGCCGGAAATACGCTGAT | TAGCGTACGTTCCCTGAGGA |
| *prg-1* | GATCAACCCATCATCATCTCTGAAGG | CAGTTTTCGGCTCTCAACAAGTC |
| *prde-1* | GCCGTGCAAGAAATTTGGGATTC | GGATTCTGACGACCATGAAGAAGAC |
| *drh-3* | TGGCCAGAAGAGCTTGAAGAAC | ACGCAATACAAGTTCAGTAACCACC |
| *hpl-2* | AAGGAGGTGAACAGTCGATACCG | TCGGTTGTAAGAATTCGATGTCCC |
| *hrde-1* | CGGCCAAGGGAGATGTCAATTC | GATCGTCACCAGTGTAGACTCC |
| *meg-1* | CGTAGTATTGGAGGCTCAGCTC | GGTTAACTCTTTGTTGTGCCAACG |
| *meg-3* | CTGACATCATCCAGCAAGTCTTCA | CTGGAATTCCGGTAATGTAAGAGC |
| *meg-4* | GAAACAGAGAAGGTCACCGCT | GATCCGTGGTGAGATCTGCC |
| *dcr-1* | AGGGACTACCAGGTGGAACTTTTG | GTGGATAGCTTGTTGTTCAACGAGG |
| *sid-1* | ACAGGATAGGCGACCCTCATTT | CGTCAGCTTCTGATTCGACAACG |
| *sid-2* | CGGCTAGCCTATTGATAAACGATGC | GAAGTATCCGGTGTAATTAGCGGT |
| *atfs-1* | AGCAGCCGTTGAAGGTAACC | GTCCAGTAGCTCGAGCTCTT |
| *dve-1* | ACAAGTGCTCGAGGCCTCATAC | TGCACAGAATTTGAATCCGTAGATGG |
| *ubl-5* | GAAATCACAGTAAACGATCGACTCGG | CAACACGATCTTTTCCCATCGTGTT |
| *wrt-1* | GATTCTAGTTTTATGGCAGGTGCTG | CACTAGCCTTGAATCCTCCAACC |
| *wrt-3* | CAATGTGCTGCGAATTTGAAGGA | GTAGTAAGCTTGTCCTTCATCATCC |
| *wrt-7* | TCCGTTCAGCTTCGAGATTCTACC | GGATATGTATATATTGGGTCGTCACG |
| *wrt-8* | AGTTCAGAACTCGACATGCGA | GTTGATTCACGAAGTGGTGCG |
| *wrt-9* | GTTATAAGCCTAAAGCGGAGTGTCC | CTTCTCCTGCTGAGATGGTGGT |
| *ptr-1* | GGAAACATTATGACATTGCCTACACCG | GCCAACCAATAGGATACGTGATG |
| *ptr-5* | CAGTCTCGGCAATGGACAACC | TCGCGTATGCGATCTTTGAGA |
| *ptr-10* | GCACCTAGTAAAACGGAACGACG | ACGATTGCTGAATTGGACTGTG |
| *ptr-12* | CGCTTCTCATGAAGCGGTCA | GTTATCATTTGGTGTAGTGGCCAC |
| *ptr-20* | CAGGCTCATAAGTTTGTCGCTG | CTGAAGAGCTTCAGCATTTGGGA |
| *che-14* | GGAGTGTTGCCAATATGTTTGCATG | TGGTAACTCTCTACACGTGGCAC |
| *gpn-1* | GATATTCACGGAGTGATGTGCTCA | GATCCATGTGAACGGTTCGCAG |
| *rib-2* | GGGAGCAGAAACGAATCCTTGC | CTCGGAGCTCTTCTAACTGTCG |
| *hhat-1* | GACCCTTGATGAAGGACGTCTCAAAC | GTATGCAACAGCTGCAATCGAG |
| *ptd-2* | AACCGACAGCTTATTTGCTGCA | GTGTGACATTCTCTCAACTTCTCTCC |
| *elt-2* | CAGTAAACGGAGGAATGATGTGCG | CAGTTGGCTGCTCTGAAGGT |
| *clec-65* | CCCGGTGGTGACTGTGAATA | AGCTCATATTGTCGCTGGCA |
| *lys-2* | ATCGACTCGAACCAAGCTGCG | TCGACAGCATTTCCCATTGAAGCGT |
| *atp-2* | GATCTCAAGGGAAAGAACTCCAAGG | GGACACTTCAGATCCAGCCTG |
| *cco-1* | CTGGAGATGATCGTTACGAGCCA | CTTGCTCACACATGCATCCAATG |
| *spg-7* | GGAAAGTATGCAGGACAAACGTGC | GCTGATGAGGTTTGGGATTTCG |
| *irg-4* | TTGTGGCAAAGGAGAGGATG | ACGGTAGATTGCTAATGGGTTC |
| *lys-7* | CGGGTTATTGTGCAGTTTTCG | AATCTCAATTCCGAGTCCAGC |
| Asns | GCAGTGTCTGAGTGCGATGAA | TCTTATCGGCTGCATTCCAAAC |
| Gli1 | AGCGTGAGCCTGAATCTGTG | CAGCATGTACTGGGCTTTGAA |
| Smo | GAAGTGCCCTTGGTTCGGA | GCAGGGTAGCGATTCGAGTT |
| Hhip | TGAAGATGCTCTCGTTTAAGCTG | CCACCACACAGGATCTCTCC |
| Gapdh | TCACCACCATGGAGAAGGC | GCTAAGCAGTTGGTGGTGCA |
| Chop | CTGGAAGCCTGGTATGAGGAT | CAGGGTCAAGAGTAGTGAAGGT |
| 21ur-9942 | GCGCGGGTTGAAGAAATTGAAGAAATTA | Universal primer (Vazyme, MR101-01) |
| 21ur-9245 | GGCGGTTTGGCTCAAAATCTGAAAAA | Universal primer (Vazyme, MR101-01) |
| 21ur-13372 | GGCCGGTAACGTGAAAAAATTGAGGA | Universal primer (Vazyme, MR101-01) |
| 21ur-10813 | GGGCCGTATTCCGTTTTTTTATATTTA | Universal primer (Vazyme, MR101-01) |
| 21ur-9641 | GGGCGCCTTTTTTCTTTTTATTTTTCA | Universal primer (Vazyme, MR101-01) |
| 21ur-6889 | GGCGAGCAATTATTTATGTCTTCCTAA | Universal primer (Vazyme, MR101-01) |
| 21ur-6059 | GCGGCTCGACAAATTCCCTTCTCACA | Universal primer (Vazyme, MR101-01) |
| 21ur-3765 | GCGGAGGCTCGGAAAATAAAATAATTAA | Universal primer (Vazyme, MR101-01) |
| 21ur-11925 | GGCAGGGAAAATCTGATAACTATGAAA | Universal primer (Vazyme, MR101-01) |
| 21ur-10851 | GGCGGCACAATGAATACGAATACAAAA | Universal primer (Vazyme, MR101-01) |
| 21ur-265 | GGGCCCGAAAATAAATAATTCCATTCAA | Universal primer (Vazyme, MR101-01) |
| 21ur-6555 | GGACGCGAGCAAATACCACGATTATA | Universal primer (Vazyme, MR101-01) |
| 21ur-5847 | CCGCCGCTCAATTCCAATACCCAATAAA | Universal primer (Vazyme, MR101-01) |
| 21ur-4659 | GGGCAGGCCCTTTTATAAAGCTGAAAATA | Universal primer (Vazyme, MR101-01) |
| 21ur-12303 | GGCCGCGGGTCCTGAAAATAAAGAAAGA | Universal primer (Vazyme, MR101-01) |
| 21ur-6464 | GGCAGGGCACAGATTTCTGAGTGATGGAA | Universal primer (Vazyme, MR101-01) |
| 21ur-32 | GGCGGGCTGTTCTAAAGATGAAGTTCTA | Universal primer (Vazyme, MR101-01) |
| U6 | CGGGAAGAGAGGCCGTGAATAGAGAG | Universal primer (Vazyme, MR101-01) |
| *tomm-20* | CGGCTACTGCATTTACTTCGA | TCATTGCCTGCTGCAGCTGGA |
| *tomm-22* | CGACTTCGTTCAGCAGTTCAT | GCGATCAATGACGTTGTAGATA |
| *tomm-40* | AGCTCGTGATGTCTTCCCAAC | TCCAAATCGGTATCCGGTGTT |
| *timm-17B.1* | GATTGTTGTCTTGTCGCCATCC | ATCACCTTTGGTCCTGAACGG |
| *timm-23* | AGTGCCGGAATGAACTTCTC | GTTGATCCAAGGCGAGGAC |
| *tin-44* | GGGATACGATTAACTCGGACA | CTGCATTCGAGCTTTCAACTG |
| *mppa-1* | CGATTTTGTGACTGTTGGCGT | GCTTGAGAACGATTCCGATGA |
| *mppb-1* | GCACAAGTTCAGCCGAAATCA | TTCTCATTCTCGTAGCGACTG |
| *wrt-10* | GTTACACCTCGTGTCGGATCTC | GGCAAGAAGTTTGATCGTCAATGC |
