# Supplementary material for: A germline-to-soma signal triggers an age-related decline of mitochondrial stress response
Source: Nat Commun. 2024 Oct 8;15:8723. doi: 10.1038/s41467-024-53064-0 (PMC11461804; doi:10.1038/s41467-024-53064-0)
Supplement: Supplementary file 7 — Supplementary Data 5 [file 41467_2024_53064_MOESM7_ESM.docx]

**Information of RNAi clones in this study**

| **RNAi clones** | **Source** | **Primer sequences** |
| --- | --- | --- |
| *wrt-5* | this study | Forward: tggatccaccggttccatggCATGTGGCTCATGGCTTCATGG  Reverse: ctcactatagggcgaattggTATTCACAACACATAAGCCGCAGAC |
| *wrt-7* | this study | Forward: tggatccaccggttccatggAATGTGTGCTTGTCGTTGCAC  Reverse: ctcactatagggcgaattggACTGCTACGTTCGGAACTTGATG |
| *wrt-8* | this study | Forward: tggatccaccggttccatggCAGATGTGGAGAATCTACGATTCCG  Reverse: ctcactatagggcgaattggGTTTGTCCATCCACAACTTCTCCG |
| *ptr-12* | this study | Forward: tggatccaccggttccatggTGACAGCCGAATCACATATCCA  Reverse: ctcactatagggcgaattggTATTTTTGGAAGACGCCCTCC |
| *che-14* | this study | Forward: tggatccaccggttccatggGATCGAACGTGTCGGTCGTCTCTA  Reverse: ctcactatagggcgaattggACAGCCACGCATCACAGAC |
| *prg-1* | this study | Forward: tggatccaccggttccatggGAGGTTCAACACCCTATCGACAGAT  Reverse: ctcactatagggcgaattggACGAGCCTCCTCAATGAACTTCT |
| *prde-1* | this study | Forward: tggatccaccggttccatggAGCAATCGTCACTTGGCTATTCTCA  Reverse: ctcactatagggcgaattggCTGTATAATGTTCGCGAGTCGCT |
| *drh-3* | this study | Forward: tggatccaccggttccatggCGATGAGAAGGGACTCAGAATTGC  Reverse: ctcactatagggcgaattggTACTTCATCACGACGTCCTGTTCCA |
| *hpl-2* | this study | Forward: tggatccaccggttccatggTGTCGAGCAAATCAACAAAGCGAG  Reverse: ctcactatagggcgaattggGAGTTTCTTGGGAACAAGAGACTGTC |
| *hrde-1* | this study | Forward: tggatccaccggttccatggCGATGGACACGATGAATGTTCAGA  Reverse: ctcactatagggcgaattggAGTGCTTCTTCAATGCATCGCTTC |
| *meg-1* | this study | Forward: tggatccaccggttccatggCAACGTCATTGGACAAACATGCTCA  Reverse: ctcactatagggcgaattggTGATGCTTGGGAGCGTCACTGA |
| *meg-3* | this study | Forward: tggatccaccggttccatggCCAACAGCTGAATCACTCGATGAC  Reverse: ctcactatagggcgaattggGGAGTCAAAGTCTTCACCAATTGAGC |
| *meg-4* | this study | Forward: tggatccaccggttccatggGATGAATGCAGCTCGAGCTAACAGA  Reverse: ctcactatagggcgaattggACGACGATGTTTCTGCACTGAAG |
| *dcr-1* | this study | Forward: tggatccaccggttccatggGCTATCCACATCGAAGTTCACACAAG  Reverse: ctcactatagggcgaattggCTGGCTCCAACGACGTAATCC |
| *sid-1* | this study | Forward: tggatccaccggttccatggACCGTGCAGTCTCGATTGAATG  Reverse: ctcactatagggcgaattggCAAGCTCGAAGTTCTCCCAATGG |
| *ptr-16* | this study | Forward: tggatccaccggttccatggGATGAGTACTACGTTGGAGAGCACT  Reverse: ctcactatagggcgaattggCGTTCGTAGATCCATGCGATATGG |
| *wrt-3* | Ahringer library | n/a |
| *wrt-6* | Ahringer library | n/a |
| *wrt-9* | Ahringer library | n/a |
| *atfs-1* | Ahringer library | n/a |
| *sid-2* | Ahringer library | n/a |
| *ptr-1* | Ahringer library | n/a |
| *ptr-5* | Ahringer library | n/a |
| *ptr-10* | Ahringer library | n/a |
| *ptr-20* | Ahringer library | n/a |
| *gpn-1* | Ahringer library | n/a |
| *rib-2* | Ahringer library | n/a |
| *hhat-1* | Ahringer library | n/a |
| *ptd-2* | Ahringer library | n/a |
| *dve-1* | Ahringer library | n/a |
| *ubl-5* | Ahringer library | n/a |
| *elt-2* | Ahringer library | n/a |
| *wrt-1* | Ahringer library | n/a |
| *wrt-10* | Ahringer library | n/a |
| *atp-2* | Ahringer library | n/a |
| *cco-1* | Ahringer library | n/a |
| *spg-7* | Ahringer library | n/a |
| *ptr-8* | Ahringer library | n/a |
