# Supplementary material for: A germline-to-soma signal triggers an age-related decline of mitochondrial stress response
Source: Nat Commun. 2024 Oct 8;15:8723. doi: 10.1038/s41467-024-53064-0 (PMC11461804; doi:10.1038/s41467-024-53064-0)
Supplement: Supplementary file 8 — Reporting Summary [file 41467_2024_53064_MOESM8_ESM.pdf]

nature portfolio

Corresponding author(s): Ying Liu

Last updated by author(s): September 13, 2024

Reporting Summary

Nature Portfolio wishes to improve the reproducibility of the work that we publish. This form provides structure for consistency and transparency in reporting. For further information on Nature Portfolio policies, see our Editorial Policies and the Editorial Policy Checklist.

Statistics

For all statistical analyses, confirm that the following items are present in the figure legend, table legend, main text, or Methods section.

n/a	Confirmed	
☐	☒	The exact sample size (*n*) for each experimental group/condition, given as a discrete number and unit of measurement
☐	☒	A statement on whether measurements were taken from distinct samples or whether the same sample was measured repeatedly
☐	☒	The statistical test(s) used AND whether they are one- or two-sided<br>*Only common tests should be described solely by name; describe more complex techniques in the Methods section.*
☒	☐	A description of all covariates tested
☐	☒	A description of any assumptions or corrections, such as tests of normality and adjustment for multiple comparisons
☐	☒	A full description of the statistical parameters including central tendency (e.g. means) or other basic estimates (e.g. regression coefficient) AND variation (e.g. standard deviation) or associated estimates of uncertainty (e.g. confidence intervals)
☐	☒	For null hypothesis testing, the test statistic (e.g. *F*, *t*, *r*) with confidence intervals, effect sizes, degrees of freedom and *P* value noted<br>*Give P values as exact values whenever suitable.*
☒	☐	For Bayesian analysis, information on the choice of priors and Markov chain Monte Carlo settings
☒	☐	For hierarchical and complex designs, identification of the appropriate level for tests and full reporting of outcomes
☐	☒	Estimates of effect sizes (e.g. Cohen's *d*, Pearson's *r*), indicating how they were calculated

Our web collection on statistics for biologists contains articles on many of the points above.

Software and code

Policy information about availability of computer code

Data collection	1, Q-PCR: Bio-Rad CFX manager, version: 3.1.1517.823<br>2, C. elegans images: AFN 2012(Bule edition), version: 1.1.13346.204<br>3, ELISA: Spectramax i3x
Data analysis	1, Statistics analysis: Graphpad Prism, version: 9.0<br>2, Statistics analysis: Image J FIJI software<br>3, Sequencing quality evaluation: FastQC, version 0.11.9<br>4, mRNA-seq data analysis: HISAT2, version 2.2.1; HTSeq, version 0.12.4; DESeq2, version 1.26.0; clusterProfiler R package ,version 4.10.0

For manuscripts utilizing custom algorithms or software that are central to the research but not yet described in published literature, software must be made available to editors and reviewers. We strongly encourage code deposition in a community repository (e.g. GitHub). See the Nature Portfolio guidelines for submitting code & software for further information.

Data

Policy information about availability of data

All manuscripts must include a data availability statement. This statement should provide the following information, where applicable:

- Accession codes, unique identifiers, or web links for publicly available datasets
- A description of any restrictions on data availability
- For clinical datasets or third party data, please ensure that the statement adheres to our policy

The RNA-seq data generated in this study have been deposited in the GEO under accession code GSE265886 (https://www.ncbi.nlm.nih.gov/gds/?term=GSE265886). All data supporting the findings of this study are available within this paper and its Supplementary Information files. Source data are provided with this paper.

Research involving human participants, their data, or biological material

Policy information about studies with human participants or human data. See also policy information about sex, gender (identity/presentation), and sexual orientation and race, ethnicity and racism.

Reporting on sex and gender	*Use the terms sex (biological attribute) and gender (shaped by social and cultural circumstances) carefully in order to avoid confusing both terms. Indicate if findings apply to only one sex or gender; describe whether sex and gender were considered in study design; whether sex and/or gender was determined based on self-reporting or assigned and methods used. Provide in the source data disaggregated sex and gender data, where this information has been collected, and if consent has been obtained for sharing of individual-level data; provide overall numbers in this Reporting Summary. Please state if this information has not been collected. Report sex- and gender-based analyses where performed, justify reasons for lack of sex- and gender-based analysis.*
Reporting on race, ethnicity, or other socially relevant groupings	*Please specify the socially constructed or socially relevant categorization variable(s) used in your manuscript and explain why they were used. Please note that such variables should not be used as proxies for other socially constructed/relevant variables (for example, race or ethnicity should not be used as a proxy for socioeconomic status). Provide clear definitions of the relevant terms used, how they were provided (by the participants/respondents, the researchers, or third parties), and the method(s) used to classify people into the different categories (e.g. self-report, census or administrative data, social media data, etc.) Please provide details about how you controlled for confounding variables in your analyses.*
Population characteristics	*Describe the covariate-relevant population characteristics of the human research participants (e.g. age, genotypic information, past and current diagnosis and treatment categories). If you filled out the behavioural & social sciences study design questions and have nothing to add here, write "See above."*
Recruitment	*Describe how participants were recruited. Outline any potential self-selection bias or other biases that may be present and how these are likely to impact results.*
Ethics oversight	*Identify the organization(s) that approved the study protocol.*

Note that full information on the approval of the study protocol must also be provided in the manuscript.

Field-specific reporting

Please select the one below that is the best fit for your research. If you are not sure, read the appropriate sections before making your selection.

☒ Life sciences ☐ Behavioural & social sciences ☐ Ecological, evolutionary & environmental sciences

For a reference copy of the document with all sections, see nature.com/documents/nr-reporting-summary-flat.pdf

Life sciences study design

All studies must disclose on these points even when the disclosure is negative.

Sample size	For worm assays, sample sizes were determined based on professional standards specific to each assay type. In survival assays, each group contained a minimum of 50 worms. For brood size assays, at least 10 worms per group were measured. GFP reporter assays involved measuring at least 20 worms per group, while in the PAO1(GFP)/PA14 CFU assays, at least 6 worms per group were assessed. For developmental stage and exploded phenotype assays, we measured at least 100 worms per group. Detailed information can be found in the figures, legends or source data files. For mouse studies, we did not pre-determine sample sizes using statistical methods, however, we ensured consistent sample sizes were used across experiments. The number of male and female mice used in each experiment is listed in the figures, legends or source data.
Data exclusions	For survival assays involving worms, individuals that crawled off the plate or experienced vulva bursting, which could lead to irregular deaths, were not included in the data analysis. For mouse assays, no data were excluded from this study.
Replication	All experiments were repeated at least three times and yielded similar results.

Randomization	For all experiments, samples, worms, and mice were randomly allocated into groups.
Blinding	During the initial RNAi screening of candidates, the names of the targeted genes were blinded. Investigators were not blinded for experimental design, conduction, data analysis, and interpretations in most cases.

Reporting for specific materials, systems and methods

We require information from authors about some types of materials, experimental systems and methods used in many studies. Here, indicate whether each material, system or method listed is relevant to your study. If you are not sure if a list item applies to your research, read the appropriate section before selecting a response.

Materials & experimental systems

n/a	Involved in the study
☐	☒ Antibodies
☐	☒ Eukaryotic cell lines
☒	☐ Palaeontology and archaeology
☐	☒ Animals and other organisms
☒	☐ Clinical data
☒	☐ Dual use research of concern
☒	☐ Plants

Methods

n/a	Involved in the study
☒	☐ ChIP-seq
☒	☐ Flow cytometry
☒	☐ MRI-based neuroimaging

Antibodies

Antibodies used	1. Rabbit polyclonal anti-Flag antibody (Sigma Cat# F7425;1:2000 dilution for immunoblotting)<br>2. Rabbit monoclonal anti-HA antibody (Cell Signaling Technology Cat# 3724; 1:2000 dilution for immunoblotting)<br>3. Rabbit monoclonal anti-b-Actin antibody (ABclonal Cat# AC026; 1:1000 dilution for immunoblotting)<br>4. Rabbit polyclonal anti-HSP60 antibody (Cell Signaling Technology Cat# 4870; 1:1000 dilution for immunoblotting)<br>5. Rat monoclonal anti-Tubulin antibody (Abcam Cat# ab6161; 1:1000 dilution for immunoblotting)<br>6. Mouse polyclonal anti-GFP antibody (Sungene Cat#KM8009; 1:2000 dilution for immunoblotting)
Validation	The antibodies used in this study were validated by the manufacturers and have been used in previously published studies.<br>1. Mouse monoclonal anti-GFP antibody: Western blot of C. elegans was validated. Citation [PMID]: 30642431.<br>2. Rat monoclonal anti-tubulin antibody: Western blots of C. elegans was validated. Citation [PMID]: 30642431,17608927.<br>3. Rabbit polyclonal anti-FLAG antibody: Western blots of 293T cells was validated. Citation [PMID]: 31645592, 28973854.<br>4. Rabbit monoclonal anti-HA antibody: Western blots of 293T cells was validated. Citation [PMID]:38019907<br>5. Rabbit monoclonal anti-b-Actin antibody: Western blots of 293T cells was validated. Citation [PMID]:37071992<br>6. Rabbit polyclonal anti-HSP60 antibody: Western blots of C. elegans was validated by RNAi knockdown.

Eukaryotic cell lines

Policy information about cell lines and Sex and Gender in Research

Cell line source(s)	HEK293T(CRL-3216) was from ATCC.
Authentication	HEK293T(CRL-3216) was verified by ATCC via short tandem repeat (STR).
Mycoplasma contamination	All cell lines were validated to be mycoplasma-negative before used for the experiments.
Commonly misidentified lines (See ICLAC register)	No commonly misidentified cell lines in ICLAC were used in this study.

Animals and other research organisms

Policy information about studies involving animals; ARRIVE guidelines recommended for reporting animal research, and Sex and Gender in Research

Laboratory animals	Worm strains were used in this study, with detailed information available in the supplementary information. Additionally, seven-month-old wild-type C57/BL6 mice were used in this study.
Wild animals	This study did not involve wild animals.
Reporting on sex	All worm assays were performed using hermaphrodites. For mouse assays, both female and male mice were included in this study.
Field-collected samples	No field collected samples were used in this study.

Ethics oversight	Mouse experiments were conducted at Peking University and adhered to the institutional guidelines set by the IACUC.

Note that full information on the approval of the study protocol must also be provided in the manuscript.

Plants

Seed stocks	n/a
Novel plant genotypes	n/a
Authentication	n/a
