# Supplementary material for: A germline-to-soma signal triggers an age-related decline of mitochondrial stress response
Source: Nat Commun. 2024 Oct 8;15:8723. doi: 10.1038/s41467-024-53064-0 (PMC11461804; doi:10.1038/s41467-024-53064-0)
Supplement: Supplementary file 9 — Source Data [file 41467_2024_53064_MOESM9_ESM.pdf]

nature portfolio

Peer Review File

A germline-to-soma signal triggers an age-related decline of mitochondrial stress response

Corresponding Author: Professor Ying Liu

This manuscript has been previously reviewed at another journal. This document only contains reviewer comments, rebuttal and decision letters for versions considered at Nature Communications. Mentions of prior referee reports have been redacted.

Version 0:

Reviewer comments:

Reviewer #1

(Remarks to the Author)
The Authors have addressed all my comments and I support publication of he paper

Reviewer #2

(Remarks to the Author)
I thank the authors for following the reviewers' suggestions and substantially improving the manuscript. One of my critical comments was about the proposition that piRNAs in the embryo lysate directly regulate wtr-5 and wtr-6 expression and how this regulation can physiologically occur during embryonic and larva development until adulthood. In support of this hypothesis, the Authors provided in the revised version of the manuscript that 1) the piRNAs predicted to target wtr-5 and wtr-6 are abundant in the embryo lysate and decrease during larval development, 2) overexpression of those piRNAs decreased the levels of wrt-5/6 in the germline and recapitulate the phenotype observed with RNAi knockdown of wrt-5/6, 3) in vitro synthesized oligos with piRNA sequences supplemented with prg-1 embryo lysates induced the UPRmt in adults.

Even if I acknowledge the efforts of identifying piRNAs in the embryo lysate that can potentially regulate wrt-5/6 in vivo, there is still a lack of experiments suggesting a direct regulation of wrt-5/6 by PIWI and endogenous piRNAs under physiological conditions. The piRNA overexpression is, in fact, a method to induce gene silencing even more efficiently than RNAi (Priyadarshini et al., 2022). This doesn't mean endogenous piRNAs detected in the embryo can induce such silencing. The same applies to introducing synthetic oligos with piRNA sequences (we don't even know if they might be loaded into PIWI or other Argonautes).

The production of 22G-RNAs, which is the result of piRNA targeting and amplification of silencing signals, coupled with the upregulation of target genes in mutants of the piRNA pathways (and loss of 22G-RNAs) will be a more effective way to prove such endogenous regulation. Even if the piRNAs potentially targeting wrt-5/6 decrease across larval development, this constitutes correlative evidence. Based on the piRTarBase tool, wrt-5 mRNA is not increased in the prg-1 mutant, and it is not clear whether 22G-RNAs are changing in the prg-1 mutant. 22G-RNAs antisense to wrt-5 are actually increased in two datasets of prg-1 mutants (Goh et al., 2012, Shen et al. 2018), not changing in another dataset (Batista et al. 2008) and decreased in Lee et al. 2012 dataset. Moreover, no 22G-RNAs are detected in all four 22G-RNA datasets for wrt-6, and there are no gene expression changes for wrt-6 in the prg-1 mutant. The authors have also reported in their Referee Figure 1 that those mRNAs are not changing in prg-1 and hrde-1 L4 mutants (data that should be available in the paper even if constitute a negative result). Therefore, it is quite challenging to interpret these results, and it will be more appropriate to quantify all those changes directly in the tissue or developmental stage in which the authors think this regulation takes place. Lastly, in Referee Figure 1d, the authors do not show significant enrichment of 22G-RNAs in HRDE-1, as they claimed in their rebuttal. These data are from a tethering experiment of HRDE-1 and not experiments of immunoprecipitation of HRDE-1. To calculate the enrichment of such 22G-RNAs in HRDE-1, the authors should look into the published dataset containing 22G-RNAs from HRDE-1 Immunoprecipitation and calculate the enrichment over input 22G-RNA levels.

In the absence of such direct proof of piRNA-directed wrt-5/6 silencing, I advise the authors to revisit all their claims in the manuscript regarding piRNAs directly regulating wrt-5/6 and discuss that no robust evidence of 22G-RNAs regulating such

transcripts is present at the moment (considering the comments above).

Minor comments on the text:
- Page 4-5, lines 93-94: “and the biogenesis of piRNA-dependent 22G-RNAs (secondary siRNAs produced when piRNAs interact with target mRNAs), such as ekl-1 and drh-3.” ekl-1 is not involved in the biogenesis of piRNA-dependent 22G-RNAs and is implicated in CSR-1 pathway, so there is no relationship with piRNA pathway. The authors should also comment on how sid-1 dsRNA transporter and not DCR-1 (a dsRNA endonuclease) is involved in this process.

Reviewer #3

(Remarks to the Author)
The revised paper by Zhou and colleagues aims describes a germline to soma mechanism by which the induction of the mitochondrial UPR becomes blunted with age. Beginning from a synthetic experiment feeding embryo lysates to adult C. elegans, the authors show an induction of mito UPR which became the genesis of the fuller study here on the role of piRNAs in the germline.

I was not an original reviewer of the manuscript at [Redacted], so my goal here was to balance the response and revisions in this version to the critique and demands of the original comprehensive review. Overall, the three original reviewers gave excellent comments and critiques and although their requests were broad and demanding in scope, the enthusiasm for the paper as a whole seemed strong.

Given the extensive revisions and re-write, and the change in destination journal, I do not think it is beneficial to ask for further experimentation. Rather my assessment has been based on whether I think the authors response to the comments were reasonable and if so, if this work is of interest and above the bar for Nature Communications without further experimental data.

A key concern of the initial review was wanting stronger support for the role of piRNAs in the proposed mechanism. Although the revised data didn’t quite get the slam dunk from the sequencing, I do think the piRNA over expression data added are a good indicator.

Overall, despite its slightly odd ‘origin story’, I think this work is a good fit for the readers of Nature Communications and has a depth of data that support its broad findings. It remains slightly unclear if this mechanism is utilized by the animal to modulate its somatic mito URR response endogenously, but the work will become the first step toward answering that question.

Textual revisions:

• The authors need to contextualize this work in the introduction/discussion in light of the recent paper by the Dillin lab “The germline coordinates mitokine signaling”. Although this paper shows a key role for the germine in induction of somatic mito UPR, I do not think this takes from the work by Zhou et al., but rather adds to this study.
• I think the paper authors should describe more fully their initial rationale for feeding adult worms embryo lysates. Serendipitous or not, there was a reason!
• Discussion of the negative sequencing data and alternate explanations beyond piRNAs should be added – specifically a response to original reviewer 2’s comment “Transcriptional activation of wrt-5 and wrt-6 genes in the germline of adult/aged worms would be enough to explain the observed phenotype.”

Open Access This Peer Review File is licensed under a Creative Commons Attribution 4.0 International License, which permits use, sharing, adaptation, distribution and reproduction in any medium or format, as long as you give appropriate credit to the original author(s) and the source, provide a link to the Creative Commons license, and indicate if changes were made.
In cases where reviewers are anonymous, credit should be given to 'Anonymous Referee' and the source.
The images or other third party material in this Peer Review File are included in the article's Creative Commons license, unless indicated otherwise in a credit line to the material. If material is not included in the article's Creative Commons license and your intended use is not permitted by statutory regulation or exceeds the permitted use, you will need to obtain permission directly from the copyright holder.
To view a copy of this license, visit https://creativecommons.org/licenses/by/4.0/

Reviewers' comments:

Reviewer #1:

The Authors have addressed all my comments and I support publication of the paper.

We thank all the reviewer for the positive remarks on our work and are particularly grateful for the constructive comments that helped us improve the study.

Reviewer #2:

I thank the authors for following the reviewers' suggestions and substantially improving the manuscript. One of my critical comments was about the proposition that piRNAs in the embryo lysate directly regulate *wtr-5* and *wtr-6* expression and how this regulation can physiologically occur during embryonic and larva development until adulthood. In support of this hypothesis, the Authors provided in the revised version of the manuscript that 1) the piRNAs predicted to target *wtr-5* and *wtr-6* are abundant in the embryo lysate and decrease during larval development, 2) overexpression of those piRNAs decreased the levels of *wrt-5/6* in the germline and recapitulate the phenotype observed with RNAi knockdown of *wrt-5/6*, 3) in vitro synthesized oligos with piRNA sequences supplemented with *prg-1* embryo lysates induced the UPR^{mt} in adults.

Even if I acknowledge the efforts of identifying piRNAs in the embryo lysate that can potentially regulate *wrt-5/6* in vivo, there is still a lack of experiments suggesting a direct regulation of *wrt-5/6* by PIWI and endogenous piRNAs under physiological conditions. The piRNA overexpression is, in fact, a method to induce gene silencing even more efficiently than RNAi (Priyadarshini et al., 2022). This doesn't mean endogenous piRNAs detected in the embryo can induce such silencing. The same applies to introducing synthetic oligos with piRNA sequences (we don't even know if they might be loaded into PIWI or other Argonautes).

The production of 22G-RNAs, which is the result of piRNA targeting and amplification of silencing signals, coupled with the upregulation of target genes in mutants of the piRNA pathways (and loss of 22G-RNAs) will be a more effective way to prove such endogenous regulation. Even if the piRNAs potentially targeting *wrt-5/6* decrease across larval development, this constitutes correlative evidence. Based on the piRTarBase tool, *wrt-5* mRNA is not increased in the *prg-1* mutant, and it is not clear whether 22G-RNAs are changing in the *prg-1* mutant. 22G-RNAs antisense to *wrt-5* are actually increased in two datasets of *prg-1* mutants (Goh et al., 2012, Shen et al. 2018), not changing in another dataset (Batista et al. 2008) and decreased in Lee et al. 2012 dataset. Moreover, no 22G-RNAs are detected in all four 22G-RNA datasets for *wrt-6*, and there are no gene expression changes for *wrt-6* in the *prg-1* mutant. The authors have also reported in their Referee Figure 1 that those mRNAs are not changing in *prg-1* and *hrde-1* L4 mutants (data that should be available in the paper even if constitute a negative result). Therefore, it is quite challenging to interpret these results, and it will be more appropriate to quantify all those changes directly in the tissue or developmental stage in which the authors think this regulation takes place.

We have followed the reviewer's recommendation and incorporated this data in Referee Figure 1 into the revised manuscript (Supplementary figure 5i). We suspect that alternative mechanisms, potentially involving epigenetic regulation, may compensate for the effects on *wrt-5/6* expression in these mutants. In the future, we could use the auxin-inducible degradation (AID) system to transiently knock down *prg-1* and then examine the changes in the relevant 22G-RNAs and *wrt-5/6* expression.

Lastly, in Referee Figure 1d, the authors do not show significant enrichment of 22G-RNAs in HRDE-1, as they claimed in their rebuttal. These data are from a tethering experiment of HRDE-1 and not experiments of immunoprecipitation of HRDE-1. To calculate the enrichment of such 22G-RNAs in HRDE-1, the authors should look into the published dataset containing 22G-RNAs from HRDE-1 Immunoprecipitation and calculate the enrichment over input 22G-RNA levels.

We have examined the HRDE-1 immunoprecipitation dataset (PMID: 38302462) and did not observe a significant enrichment of 22G-RNAs specifically targeting *wrt-5/6*.

In the absence of such direct proof of piRNA-directed wrt-5/6 silencing, I advise the authors to revisit all their claims in the manuscript regarding piRNAs directly regulating wrt-5/6 and discuss that no robust evidence of 22G-RNAs regulating such transcripts is present at the moment (considering the comments above).

We have followed the reviewer and editor's recommendations by revising our claims in the manuscript, softening the assertions regarding piRNAs directly regulating *wrt-5/6*. Additionally, we have included a discussion acknowledging the current lack of robust evidence for 22G-RNAs regulating these transcripts (Lines 381-387).

Minor comments on the text:

- Page 4-5, lines 93-94: "and the biogenesis of piRNA-dependent 22G-RNAs (secondary siRNAs produced when piRNAs interact with target mRNAs), such as ekl-1 and drh-3." *ekl-1* is not involved in the biogenesis of piRNA-dependent 22G-RNAs and is implicated in CSR-1 pathway, so there is no relationship with piRNA pathway.

We appreciate the reviewer's insightful comment. We have removed the reference to *ekl-1* and retained only *drh-3* in the revised manuscript.

The authors should also comment on how *sid-1* dsRNA transporter and not DCR-1 (a dsRNA endonuclease) is involved in this process.

We appreciate the reviewer's insightful comment. The involvement of *sid-1*, a dsRNA transporter, rather than DCR-1, a dsRNA endonuclease, in this process may suggest that the uptake and transport of dsRNA, rather than its direct cleavage, play a more critical role in this particular pathway. We will explore this further in future experiments to clarify the distinct roles of these factors in the process.

Reviewer #3:

The revised paper by Zhou and colleagues aims describes a germline to soma mechanism by which the induction of the mitochondrial UPR becomes blunted with age. Beginning from a synthetic experiment feeding embryo lysates to adult *C. elegans*, the authors show an induction of mito UPR which became the genesis of the fuller study here on the role of piRNAs in the germline.

I was not an original reviewer of the manuscript at [Redacted], so my goal here was to balance the response and revisions in this version to the critique and demands of the original comprehensive review. Overall, the three original reviewers gave excellent comments and critiques and although their requests were broad and demanding in scope, the enthusiasm for the paper as a whole seemed strong.

Given the extensive revisions and re-write, and the change in destination journal, I do not think it is

beneficial to ask for further experimentation. Rather my assessment has been based on whether I think the authors response to the comments were reasonable and if so, if this work is of interest and above the bar for Nature Communications without further experimental data.

A key concern of the initial review was wanting stronger support for the role of piRNAs in the proposed mechanism. Although the revised data didn't quite get the slam dunk from the sequencing, I do think the piRNA over expression data added are a good indicator.

Overall, despite its slightly odd 'origin story', I think this work is a good fit for the readers of Nature Communications and has a depth of data that support its broad findings. It remains slightly unclear if this mechanism is utilized by the animal to modulate its somatic mito URR response endogenously, but the work will become the first step toward answering that question.

Textual revisions:

• The authors need to contextualize this work in the introduction/discussion in light of the recent paper by the Dillin lab "The germline coordinates mitokine signaling". Although this paper shows a key role for the germine in induction of somatic mito UPR, I do not think this takes from the work by Zhou et al., but rather adds to this study.

We appreciate the reviewer's suggestion and have incorporated a discussion of the recently published work in the manuscript.

• I think the paper authors should describe more fully their initial rationale for feeding adult worms embryo lysates. Serendipitous or not, there was a reason!

We appreciate the reviewer's suggestion and have included the rationale in the revised manuscript "Inspired by previous studies demonstrating that establishing a shared circulatory system between young and aged mice can reverse specific aging phenotypes in older animals through factors present in young mice's blood, we fed *C. elegans* embryo lysates to adult worms to explore similar effects."

• Discussion of the negative sequencing data and alternate explanations beyond piRNAs should be added – specifically a response to original reviewer 2's comment "Transcriptional activation of *wrt-5* and *wrt-6* genes in the germline of adult/aged worms would be enough to explain the observed phenotype."

We have followed the reviewer's suggestion and added a discussion in the revised manuscript regarding the negative sequencing data and alternative explanations beyond piRNAs (Lines 381-387).
